# Taking Perspective: Personal Pronouns Affect Experiential Aspects of Literary Reading

**DOI:** 10.1371/journal.pone.0154732

**Published:** 2016-05-18

**Authors:** Franziska Hartung, Michael Burke, Peter Hagoort, Roel M. Willems

**Affiliations:** 1 Neurobiology of Language, Donders Institute for Brain, Cognition, and Behaviour, Radboud University, Nijmegen, The Netherlands; 2 Neurobiology of Language, Max Planck Institute for Psycholinguistics, Nijmegen, The Netherlands; 3 Rhetoric & Argumentation, University College Roosevelt, Middelburg, The Netherlands; 4 Faculty of Humanities, Utrecht University, Utrecht, The Netherlands; 5 Centre for Language Studies, Faculty of Humanities, Radboud University, Nijmegen, The Netherlands; University of Leicester, UNITED KINGDOM

## Abstract

Personal pronouns have been shown to influence cognitive perspective taking during comprehension. Studies using single sentences found that 3^rd^ person pronouns facilitate the construction of a mental model from an observer’s perspective, whereas 2^nd^ person pronouns support an actor’s perspective. The direction of the effect for 1^st^ person pronouns seems to depend on the situational context. In the present study, we investigated how personal pronouns influence discourse comprehension when people read fiction stories and if this has consequences for affective components like emotion during reading or appreciation of the story. We wanted to find out if personal pronouns affect immersion and arousal, as well as appreciation of fiction. In a natural reading paradigm, we measured electrodermal activity and story immersion, while participants read literary stories with 1^st^ and 3^rd^ person pronouns referring to the protagonist. In addition, participants rated and ranked the stories for appreciation. Our results show that stories with 1^st^ person pronouns lead to higher immersion. Two factors—*transportation into the story world* and *mental imagery* during reading—in particular showed higher scores for 1^st^ person as compared to 3^rd^ person pronoun stories. In contrast, arousal as measured by electrodermal activity seemed tentatively higher for 3^rd^ person pronoun stories. The two measures of appreciation were not affected by the pronoun manipulation. Our findings underscore the importance of perspective for language processing, and additionally show which aspects of the narrative experience are influenced by a change in perspective.

## Introduction

Reading is a complex human behaviour in which several cognitive processes are involved. An elementary part of story comprehension is building a mental representation of the semantic contents of the text [1]. Stories, as compared to non-narrative texts, often cause the reader to get immersed into the story and construct multimodal situation models [2]. Immersion is a state of absorption, which overlaps conceptually with flow [3], and transportation [4]. These terms describe a state of absorption marked by ‘deep concentration, losing awareness of one’s self, one’s surroundings and track of time’([5], p. 28; see also [3]). Being immersed in a story is linked to mental simulation [6–9], and defined as 'the state of feeling cognitively, emotionally, and imaginally immersed in a narrative world' [4], see also [10–12], see also [13] on ‘disportation’. Immersion is also associated with enjoyment [14,11], meaning that the more we engage with a story, the more we enjoy it.

Immersion is a multidimensional experience based on factors, whose contribution to the experience of being immersed varies with the situation. Factors which often reoccur in notions of immersion in narratives include the experience of mental imagery, emotional engagement with protagonists, transportation into the story world, and attention during reading [5,15]. Experiencing imagery during narrative engagement such as mental visualizations of surroundings, characters, and situations has been hypothesized to influence immersion [5,12]. Emotional engagement with fictional characters of stories such as feelings of sympathy, empathy, and identification can facilitate immersion [5,14]. Another important factor for immersion is attention. A high level of attention towards the story is often marked by a subjective experience of losing self-awareness, awareness of the surroundings, and track of time [5]. The factor transportation ‘signifies a feeling of entering a story world, without completely losing contact with the actual world’, thus the feeling of actually being part of a fictional world during reading [5] p. 31. Transportation into a fictional world is linked to increased affective responses and identification with fictional characters [4]. In research with narratives, ‘transportation’ is also sometimes used to describe the general state of immersion into the narrative. In the present article, we treat transportation as one factor contributing to the general state of immersion or absorption during reading.

Readers can get immersed in a story by either taking the role of an observer (3^rd^ person perspective) or by taking the viewpoint of one of the characters (1^st^ person perspective) [16–18]. Readers often take the mental perspective of the protagonist and simulate his or her mental states as the point of view when constructing a situation model [19,20]. It has further been shown that with which character the viewpoint is aligned affects if readers take a 1^st^ person perspective [21]. Perspective taking is considered important in the construction and comprehension of fiction [17,22–24], and the generation of situation models [25,26]. But perspective taking is also an important topic of research in the cognitive sciences in general. Typically, perspective taking is investigated in the framework of spatial cognition see e.g.[27,28] or social cognition [29]. We assume that narrative comprehension involves both types of perspective taking, because stories include information about actions, location changes and characters.

Taking the viewpoint of a character is linked to identification: it is believed that the reader is more engaged when taking a character’s viewpoint and adopting the character’s goals and intentions. During the course of the story this results in experiencing emotions of empathy [18]. Indeed, adopting a protagonist’s perspective causes changes in the mental and emotional states of the reader [10,12,30] and this effect has been shown to be linked to story immersion [4]. Experimental evidence shows that changing narrative viewpoints leads to changes in mental viewpoints. For example, in a discourse comprehension study Black and colleagues [31] showed that participants are sensitive to consistency violations in narrative viewpoints. They show that verbal deixis in sentences like ‘[…] two men *came* in’ versus ‘[…] two men *went* in’ leads to slower reading times and decreased comprehensibility if it does not match the narrative viewpoint established by the previous context. Also, people tend to correct those inconsistencies in memory tasks [31].

The most direct means of guiding the reader to take the role of a spectator or character are *narrative perspective* and *narrative viewpoint*, e.g. [32]. Narrative perspective (Who is telling the story?) and narrative viewpoint (Whose viewpoint is the narrative constructed from?) are typically aligned with a character (or a narrator), whose mental or visual response to the events in the story is the source of construal of the narrative events for the reader [33]. Using narrative perspective, story writers can make readers 'see' through the eyes of one of the characters or take a mere spectator's view. A well-established way to guide cognitive perspective taking in text is the choice of personal pronouns, which refer to protagonists [17,34–37]. Experimental research with single sentences shows that personal pronouns in thematic agent's positions affect the spatial representation in the reader, e.g. people react faster to a picture showing tomato slicing from 1^st^ person perspective after hearing the sentence 'I am slicing a tomato', than after hearing the sentence 'he is slicing a tomato' and vice versa [35]. In a series of experiments, it has been shown that 3^rd^ person pronouns (*he*, *she*, *it*) robustly promote a 3^rd^ person perspective mental representation, whereas 1^st^ person pronouns can promote either 1^st^ person or 3^rd^ person mental perspective, depending on the contextual embedding. Prevalence for 1^st^ person perspective taking is strongest when participants are addressed directly with 2^nd^ person pronouns (e.g. *You are slicing tomatoes*.), where embodying emotional states of fictional characters is also stronger compared to other pronoun types [36]. In accordance with this, Papeo and colleagues [38] showed that only 1^st^ person action sentences show a motor simulation effect, whereas 3^rd^ person sentences do not. Moreover, neuroscientific evidence suggests that motor imagery in 1^st^ person and 3^rd^ person perspective relies on different neuronal networks [39,40].

However, despite the substantial body of narrative theory and experimental evidence from psychological studies with personal pronouns, it remains unclear how the choice of personal pronouns influences experiential aspects of literary reading such as immersion and appreciation of a story. In the present study we investigated how story immersion is affected by choice of pronoun referring to the main character. We manipulated whether literary stories were written in 1^st^ or 3^rd^ person viewpoint, that is, by using 1^st^ or 3^rd^ person pronouns referring to the protagonist. We refrained from testing second person perspective, because 2^nd^ person perspective narration is very uncommon in literary fiction, and the type of fiction in which it finds application is very different from typical 1^st^ or 3^rd^ person narration texts. This would not only limit our choice of appropriate stimulus materials substantially, but would also result in asymmetry regarding the amount of prior exposure our sample population has with the types of texts. Moreover, it has been shown that 2^nd^ person pronouns tend to be interpreted in a generic meaning, particularly in descriptive language [41]. In the present study, we combined measuring Electrodermal Activity (EDA) with appreciation ratings and established questionnaires for narrative engagement, to investigate if and how arousal, immersion, and affective responses to reading fiction are affected by choice of personal pronouns referring to protagonists. The main reason to include the EDA measure was to have a more objective, but also an online measure of arousal during the actual stimulus exposure, to relate to the self-report measures which were taken after exposure. EDA measures arousal, that is, the physical and psychological state of being alert and ready to react, which can be related to emotional stimulation, increased mental workload, and the startle reflex [42–44]. High levels of arousal lead to increased heart rate and blood pressure, sensory alertness, and sweat gland activity. EDA measures electrical conductivity in the skin, which is sensitive to changes in blood pressure and sweat production. Spontaneous increases in conductivity reflect sudden increases in arousal level as a consequence of stimulation resulting in (negative) emotion, surprise or difficulty in processing [42–44].

Following the assumption that 1^st^ person perspective facilitates a more immediate experience and therefore identification [18,38], we expect that readers are more emotionally affected by 1^st^ person perspective narratives and experience higher levels of immersion. This should result in higher scores on the immersion questionnaires, especially on the subscales for emotional engagement, transportation, and attention. Also physical arousal could be affected by the immediateness of 1^st^ person narration, because of higher suspense or emotional responses during reading. Therefore we expect that both immersion as a self-report measured by questionnaire response, as well as arousal as measured by EDA are higher when participants read 1^st^ person perspective narratives. We further expect that higher immersion results in higher appreciation [14,11], but without clear expectation as to which components of immersion might cause this effect. Moreover, we expected high individual variability regarding experiential aspects of literary reading and sensitivity to stylistic features. To be able to take this into account we measured participants’ self-reported reading behaviour, previous print exposure, and empathy. The latter is expected to correlate with immersion, print exposure and reading behaviour, following previous research which argues for a positive link between empathy and fiction reading [45–50]. We had no clear expectations towards how reading behaviour and print exposure relate to immersion, arousal during reading, or appreciation.

## Methods

In line with guidelines for psychological research, we report how we determined our sample size, all data exclusions, all manipulations, and all measures in the study.

### Participants

64 participants were recruited from the Max Planck Institute (MPI) participant database (35 female, 29 male; mean age 21.7 years, s.d. = 3.5 range 18–34). Participants were native speakers of Dutch with normal or corrected to normal vision, and no reading impairments. We asked participants for their academic history to ensure that they had no high level of experience in literature analysis. Participants were naïve as to the purpose of the experiment. Participation was voluntary and participants received money for participation. All participants gave written informed consent in accordance with the declaration of Helsinki. The study was approved by the local Ethics Committee of the Social Sciences faculty of the Radboud University (Ethics Approval Number ECG2013-1308-120). After data exclusion (see below), the data of 52 participants went into the final analysis (30 female, 22 male; mean age 21.4, s.d. = 3.2, range 18–32).

### Data exclusion

One participant stated that they had realized the manipulation of the stories during debriefing and therefore was excluded from processing. Another participant was excluded, because claimed that they were an expert in fiction writing as well as being a published author. Data from two other participants were not processed because the signal quality of the electrodermal activity (EDA) differed substantially between the two experimental blocks. Six additional participants did not enter the analysis because they did not meet the predefined minimum correctness criterion for content questions (> 25% incorrect), which we took as an indication that those participants did not pay enough attention to the content of the stories. One more participant was excluded for being an outlier, because the difference in number of peaks in the EDA between the two conditions was more than four standard deviations from the mean difference of all participants. Removing this data set left us with N = 53, so the last tested participant in the opposite order of conditions was removed to have an equal number of participants in both orders of conditions. In total, 12 participants were excluded from the analysis. All reported results are for N = 52.

### Material

#### Stories

We selected 8 short stories from Dutch fiction, which were published between 1974 and 2010 (see Table 1; mean number of words per story = 1043.25, s.d. = 723.05, range 338–2090, story summaries can be found in S1 Story Summaries and Content Questions). The selected stories were all typical short stories focusing on a single incident, had a single plot, a single setting, and covered only a short period of time. Also, there was—if at all—only a brief introduction, an open ending, and the number of characters in the story was limited. The stories were written in a laconic style avoiding direct statements of judgments and attitudes, e.g. the following excerpt from the ending of Officina Asmara (see S1 Story example A for the full transcript of the story in English and S2 Story example B and S3 Story example C for other more stories. For copyright reasons, only translations of the Dutch stories are published along with the manuscript. Please contact the corresponding author for a copy of the original stories.):

“[Son, from who’s viewpoint the story is told, asks his father] 'So now you’re a criminal?'

[Father replies] ‘Who gives a damn about the law?’

Later that evening we sit at the kitchen table in the grey light. My father's face is full of shadows. I examine this dubious man, who refurbishes old ship models in his barn between piles of paper, and resolve to take a closer look as long as time still allows.”

**Table 1:** Story Information.

Title	Author	Original perspective	Number of words	Publication year
*Rivier (River)*	Tommy Wieringa	3^rd^ person	339	2010
De Mexicaanse hond *(The Mexican dog)*	Marga Minco	1^st^ person	1239	1990
Dubbele tong *(Double tongue)*	Bernlef	3^rd^ person	2005	1974
Broeder P. *(Brother P*.*)*	Tommy Wieringa	3^rd^ person	350	2010
De tekening *(The drawing)*	Thomas Rosenboom	1^st^ person	1283	2006
De vissers *(The fishermen)*	Thomas Rosenboom	3^rd^ person	2092	2006
Liberty Mountain *(Liberty Mountain)*	Sylvia Witteman	1^st^ person	652	2009
Officina Asmara *(Officina Asmara)*	Tommy Wieringa	1^st^ person	402	2010

Eight short stories from Dutch fiction published between 1974 and 2010, were selected as stimulus material (mean number of words per story = 1043.25, s.d. = 723.05, min = 338, max = 2090). The stories were all typical short stories with a single plot, a single setting and focusing on a single incident covering only a short period of time. There was only a very brief introduction (if at all) and an abrupt and open ending. All stories were internally focalized by the main character and the narrative voice was identical with the narrative point of view. Besides the main character, the number of active characters was very limited. In the original version half of the stories used 1^st^ person pronouns to refer to the main character and half 3^rd^ person pronouns. Word count is based on the original versions of the stories.

For all stories the narrative voice was identical with the narrative point of view. All stories were internally focalized, which means that the style of narration reflects the subjective perception of the main character [23,24]. Half of the stories were referring to the protagonist with 1^st^ and half with 3^rd^ person pronouns in the original version. To make exact comparisons we created a second version of each story in the corresponding condition. This was done by changing personal pronouns and their respective verb forms (see Table 2). In addition, direct speech was changed to indirect speech for the 1^st^ to 3^rd^ person condition to support a natural reading flow in cases where direct speech seemed very unnatural as judged by a native speaker of Dutch (total number of changes made = 8 out of 98 direct speech segments).

**Table 2:** Illustration of story modification.

1^st^ person perspective (original)	3^rd^ person perspective
Ik kende Marianne nog maar kort. We waren met de veerpont overgestoken naar de havenpier, waar wij de nieuwbouw bekeken en toen een café vonden. Achterin, op een verhoog, was nog een tafeltje vrij; het liep tegen vijven, schemeruur; de kleine kaart, waarboven 'Tapas' stond, vermeldde Italiaanse paté, en vervuld van daadvaardig geluk wrong ik mij naar de toog om te bestellen. 'Twee broodjes alstublieft met. . .'	Hij kende Marianne nog maar kort. Ze waren met de veerpont overgestoken naar de havenpier, waar zij de nieuwbouw bekeken en toen een café vonden. Achterin, op een verhoog, was nog een tafeltje vrij; het liep tegen vijven, schemeruur; de kleine kaart, waarboven 'Tapas' stond, vermeldde Italiaanse paté, en vervuld van daadvaardig geluk wrong hij zich naar de toog om te bestellen. 'Twee broodjes alstublieft met. . .'
*I only had known Marianne for a short time*. *Together we took the ferry to the harbour pier*, *where we looked at the new constructions and entered a coffee bar*. *Inside at the back*, *on a little platform*, *we found a free table; it was almost 5 already*, *gloaming time; the little menu*, *with ‘Tapas’ written on the top*, *listed Italian pastries*, *and vigorously I wrestled my way to the bar*: *‘Two sandwiches please*.* *.* *.*’*	*He only had known Marianne for a short time*. *Together they took the ferry to the harbour pier*, *where they looked at the new constructions and entered a coffee bar*. *Inside at the back*, *on a little platform*, *they found a free table; it was almost 5 already*, *gloaming time; the little menu*, *with ‘Tapas’ written on the top*, *listed Italian pastries*, *and vigorously he wrestled his way to the bar*: *‘Two sandwiches please*.* *.* *.*’*

For each story a second version was created by replacing the personal pronouns referring to the main character and its related verb in each text with the personal pronoun in the corresponding condition. Example taken from *De tekening* by Thomas Rosenboom. No authorized translation is available; the current translation is for illustration purposes only.

#### Questionnaires for measuring individual differences

For an estimate of print exposure we used a Dutch version of the Author Recognition Test (ART) [48,51] containing 42 names of which 30 are existent fiction authors and 12 made up names (see S1 Author Recognition Test). In the ART, participants are instructed to read a list of names and indicate which of the writers they know. The score of each participant is computed by subtracting the sum of all incorrect answers from the sum of all correct answers. Total score can vary between -12 (only non-existent author names selected) to 30 (all correct names selected).

In addition, a **general reading habits questionnaire** was used consisting of 4 items (2 questions addressing amount and frequency of reading for pleasure, 2 questions about genre preferences; RH 1–4 in S1 Statistical Models) supplemented by the 6 items from the **fantasy scale** of the **Interpersonal Reactivity Index** (**IRI**) [52]. IRI is a self-report measure of individual differences in empathy, consisting of 4 subscales. The Fantasy scale of the IRI tests individual readiness to get transported imaginatively into the feelings and actions of fictive characters in books, movies, and plays. For the 6 items from the IRI Fantasy scale we used a 7-point scale ranging from ‘I totally agree’ (= 7) to ‘I totally disagree’ (= 1). The items of the reading habit questionnaire consisted of ‘How often do you read fiction?’ with five possible answers ranging from ‘daily’ to ‘never’, ‘How many books do you read per year?’ also with five possible answers ranging from zero to ‘more than 1 per month’, ‘Which type of fiction do you prefer?’ with 5 options including ‘prose’, ‘comic’, ‘poetry’, ‘drama’ and ‘I don’t like fiction at all’, and finally a list of 22 popular genres (e.g. ‘horror’, ‘romance’) on which subjects were asked to indicate which they like without number limitations. There was also an option to add genres which were not suggested.

As evidence from recent research suggests a positive relation of fiction reading with social factors such as empathy, interpersonal relations, and social competence (see 12, 39–44), we included the **Empathy Quotient questionnaire** to measure individual differences in empathy (**EQ**; standardized Dutch version http://www.autismresearchcentre.com/arc_tests) [53].

#### Questionnaires for main measures

The immersion questionnaire we used was based on the story world absorption scale (SWAS, [15]) and selected items from the 30-item version of the narrative engagement questionnaire (NEQ) developed by Buselle and Bilandzic [14].We used the attention, transportation, emotional engagement, and mental imagery subscales from SWAS and in addition the narrative understanding subscale from NEQ, as this is not covered by SWAS. Our questionnaire comprised of 34 items (see **S1 Immersion questionnaire**). Participants responded to the items on a 7-point Likert scale ranging from 'I totally disagree’ (1) to 'I totally agree' (7).

**Appreciation** was measured in two ways. First appreciation directly after reading each story (**Rating**) was measured by asking the participants to indicate how much they liked the story on a 10-point scale (1 = bad, 10 = brilliant). The exact wording was ‘Wat vind je van dit verhaal?’ (What do you think of this story?). For the second appreciation measure (**Ranking**) participants were provided with a list of titles of the stories and were asked to rank them in order of appreciation with the one they liked the most on top and the one they like the least at the bottom.

To test whether participants paid attention to each story, we prepared 1 content question per story, which participants answered in a multiple choice task with 3–4 alternatives of which only one was correct (see S1 Story Summaries and Content Questions). Each question indicated clearly to which story it belonged. Participants who answered more than 25% of questions incorrectly were excluded from the analysis.

### Procedure

Participants were seated in a soundproof testing cabin with a bright ceiling light, a desk lamp with two brightness levels, and a window with blinds. They were encouraged to adjust the light to personal preference and to make themselves as comfortable as possible sitting at a desk with a stable chair. The aim was to create a relaxed atmosphere with a natural reading situation. After explaining the cycle of tasks, participants gave written informed consent and the EDA sensor was attached (for details see below).

The experiment was pen and paper based. To make relevant comments and set markers in the recording file of the EDA, participants were asked to indicate every time they started and finished reading a story.

A practice trial was performed with one story to familiarize participants with the setting and order of events within a trial. The story from the practice trial was not used in the main part of the experiment. The practice task took about 10 minutes, leaving the EDA sensor time to adjust to body temperature.

The experiment was conducted as a block design consisting of 2 blocks, with 4 stories per block. The block design was chosen to avoid potential switching costs between the two perspectives. There was no repetition of story per participant: each participant read every story only once, meaning that they read eight different stories in total. Within each block participants were presented with stories in one condition, either 1^st^ or 3^rd^ person pronouns referring to the main character. Both blocks took place consecutively with 10 minutes break in between. Block order was counterbalanced across participants. Participants rated every story for appreciation and completed the Immersion Questionnaire directly after reading of each story. The stories were presented in black font (Calibri, 14pt.) on white paper (A4, landscape orientation, 2 pages per sheet, printed one sided).

After reading all stories, participants ranked the stories for appreciation and answered the content questions. This was followed by the general reading habits, ART, and EQ questionnaires.

Once participants finished the experiment, they were asked what they thought the experiment was about and whether they recognized anything specific about the selected stories or a significant change between the two blocks. This was followed by a verbal debriefing, which informed them about the research question, the experiment, and our expectations. The entire experiment took approximately 90 minutes.

### Data acquisition

We measured EDA with BrainAMP ExG MR, Acceleration Sensor (Brain Products, www.brainproducts.com), and Ag/AgCI sensor electrodes (Model F-EGSR, Grass Technologies). The signal was recorded with Brain Vision Recorder (Brain Products) at a sampling rate of 5000Hz for the first 8 participants and 1000Hz for all others, with low cut-off DC and high cut-off 1000Hz. The reason for decreasing the sampling rate was to reduce unnecessary memory requirements and processing time in the data analysis as we were not interested in high-frequency components of the EDA signal. No other filters were applied to the signal. Sensor electrodes for EDA were placed at the middle phalanx of the index and middle finger of the non-dominant hand (right hand for 4 people).

Questionnaire data were acquired with pen and paper, and later digitized manually.

### Data analysis

#### EDA

EDA signal processing was done with Matlab R2013a (MathWorks, Natick, MA, USA). The data were segmented into individual trials. Each trial was defined from the onset to the offset of reading a story. Trials with recording errors (e.g. data not saved to disk) were replaced with NaNs (out of 416 trials 13 were missing, meaning 3.1% of missing values). Linear trend was removed from time courses (‘de-trending’) and data were resampled to 100 Hz. To correct for the time at the beginning or end of each trial when participant’s movement tended to create artefacts in the EDA signal, we removed three seconds from the beginning and end of each trial.

Number spontaneous fluctuation in amplitude were computed using a peak detection algorithm in which peaks are defined as local maxima surrounded by valleys (Eli Billauer, 3.4.05, see http://www.billauer.co.il/peakdet.html; d = 0.15). This algorithm picks out peaks very well, across a range of settings. We used the number of peaks for statistical comparisons, because they reflect spontaneous fluctuations, due to increased arousal [43]. We ignored valleys in the analysis because only little is known about local minima in arousal besides habituation effects. Analysing the number of peaks in EDA is not a standard measure such as area under the curve or absolute amplitude changes relative to a baseline. Because of our experimental design which focused on the naturalness of the reading situation, the trials are relatively long and differ substantially in length. This means that we cannot time lock the EDA response to certain events which would be crucial for types of analysis based on amplitude or amplitude dependent measures.

#### Questionnaire Data

There were 87 missing values in total (0.67% of all responses), which occurred when a participant did not tick the scale for one item or when the marking was ambiguous. Missing values were replaced with the variable mean. Data points were averaged for each subscale to compute mean scores for attention, transportation, emotional engagement and mental imagery (SWAS), and narrative understanding (NEQ).

The content questions were checked for correctness. Two items were answered incorrectly by more than 25% of participants indicating unexpected difficulty and were therefore not included in the evaluation. For the remaining 6 questions we defined that more than 1 incorrect answer (= 33.33% or more) led to exclusion of the participant.

The items of the general reading habits questionnaires, and the mean scores of the Fantasy scale, EQ and ART were treated as measures of individual differences.

#### Statistical Model

All data were analysed using the statistical software package RStudio v00.96.331 (R Core Team, 2009), using the nlme library for testing linear mixed models [54]. The use of a linear mixed model allows for the inclusion of both participants and stories as random effects [55]. Each of the main measures (Immersion, Rating, Ranking, Peaks) was analysed in a separate model. First, a simple model was constructed to predict each main measure, in which the dependent variable was on the intercept, and order of conditions, pronoun type, and whether the stories were the original or the modified version were used as fixed effects. Story and participant were included as random effects in all models. In addition, a variation of this model including random slopes for participants and stories was constructed. A model comparison between the model only including random intercepts and the model including random intercepts and slopes was used to select the model with the better fit to the data. For each main measure, we constructed a second model to which we added individual differences measures, namely gender, ART score, EQ score, the score from the IRI Fantasy scale and the four question responses regarding reading habits. All numerical predictors for individual differences (EQ, ART) were centred. To test for correlations between the dependent measures, we constructed an additional model for the EDA and the appreciation measures with adding the other dependent variables as factors. In order to rule out that differences in the number of peaks between conditions are a result of different reading times in both conditions, we constructed a control model for the analysis of EDA data. The model was identical to the statistical model used for the analysis of number of peaks, with ‘duration’ as dependent variable instead of peaks.

P-values for specific effects were obtained by a model comparison procedure with asymptotic chi square distribution. We only used the full model including individual differences for exploring the subscales of the immersion questionnaire. The models and the original code can be found in S1 Statistical Models.

## Results

Here we report the results of the following main measures: Immersion, Rating, Ranking, and EDA. In addition, as immersion is a multidimensional concept, we analysed the standardized subscales of the immersion questionnaire separately in order to get a better understanding of which factors of immersion are affected by pronoun type, e.g. it is more likely that subscales directly related to the protagonist are more sensitive with regards to the main manipulation. Finally, we relate individual difference measures including EQ, ART, reading habits, and the score on the fantasy scale of the IRI to the main measure to explore their contribution to explain the variance.

### Individual Difference measures

Participants scored on the **EQ** (Empathy Quotient) questionnaire within the normal range and distribution on the standardized EQ (mean = 40.50, sd. = 11.69, min = 17, max = 63).

In the **ART** (Author Recognition Test) questionnaire, participants scored on average 6.50 writers (out of maximal 30; sd. = 4.30, min = 0, max = 22).

On the **general reading habits** items, participants indicated that they read on average once per week, ranging from daily to never (48.1% don’t read regularly, 5.8% never read, 17.3% read once per week, 21.2% read more than twice per week, and 7.7% read daily). Most participants read 3–10 books per year (34.6%), 26.9% read less than 3, 1.9% does not read at all, and 13.5% read at least 1 book per month (23.1% more than that).Regarding literature type preferences, 78.8% of participants indicated that they prefer prose. On average, participants checked 5.7 genres (s.d. = 2.02, minimum = 3, maximum = 11). On the **Fantasy scale**, participants scored on average 4.7 (s.d. = 0.81, minimum = 3.00, maximum = 6.83).

### Immersion

**Fig 1:**
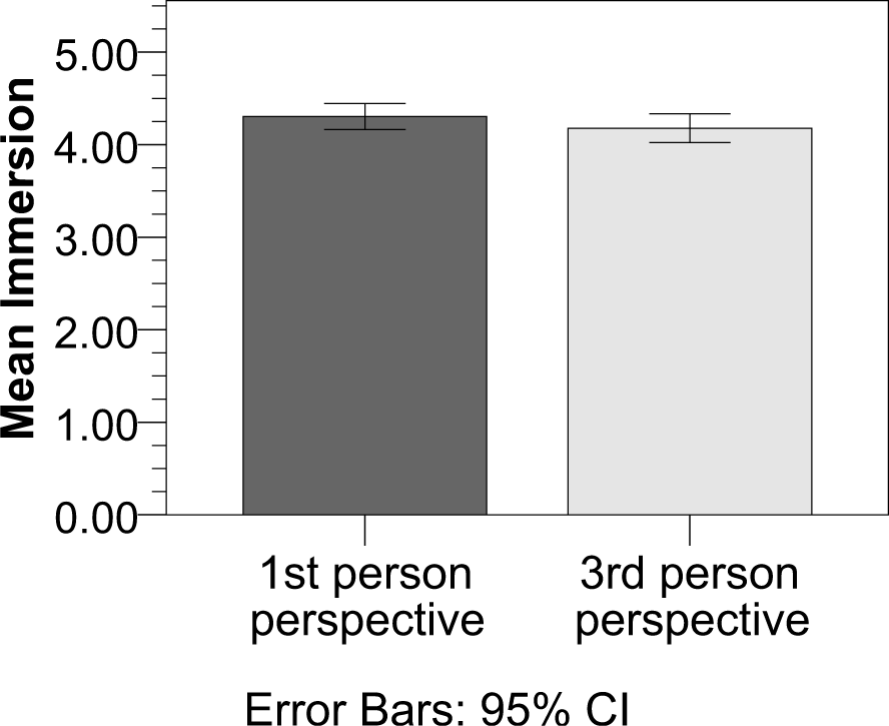
Immersion scores in stories with 1^st^ and 3^rd^ person pronouns referring to the protagonist. Participants on average scored higher on the immersion questionnaire when reading 1^st^ person pronoun narratives compared to 3^rd^ person pronoun narratives. Error bars represent 95% confidence intervals.

We first report the results of averaging over all questions in the immersion questionnaire. The findings for separate subscales follow below. The best model fit was produced when only including random intercepts for *Immersion*. Stories with 3^rd^ person pronouns showed lower scores on the immersion questionnaire than stories with 1^st^ person pronouns (β = -0.16, s.e. = 0.08, t = -1.98, p < 0.05, see Fig 1, see S1 Statistical Models: *modela2*). From all individual difference measures, only EQ contributed significantly to the model (β = 0.022, s.e. = 0.01, t = 2.57, p < 0.05), meaning a higher EQ score predicts a higher immersion score (see S1 Statistical Models *modelFULLimmersion*).

The best model fit was produced when only including random intercepts for the **attention subscale** of the immersion questionnaire. The model did not show an effect of *pronoun type* (β = -0.14, s.e. = 0.10, t = -1.45, p = 0.15, see). The EQ score however, explains a significant part of the attention scores likewise as for the overall immersion scores (β = 0.02, s.e. = 0.01, t = 2.03, p < 0.05, see S1 Statistical Models *modelATT*), meaning that a higher EQ predicts higher levels of attention during reading.

**Fig 2:**
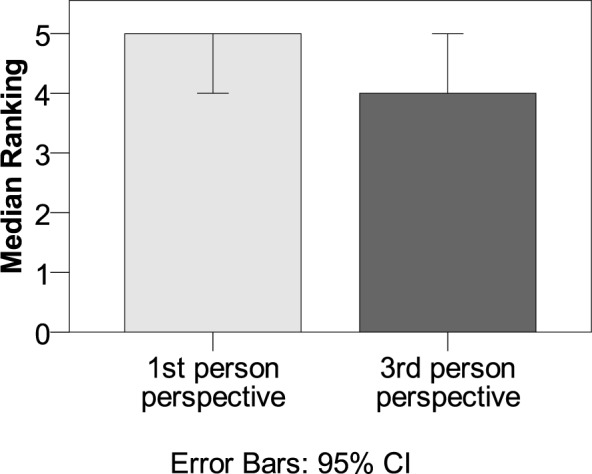
Subscales of the immersion questionnaire. The subscales were emotional engagement, narrative understanding, transportation, attention, and mental imagery. Differences between stories with 1^st^ and 3^rd^ person pronouns referring to the protagonist were significant for the transportation and the mental imagery subscale.

The best model fit was produced when only including random intercepts for the **transportation subscale.** Here, we observe an effect of *pronoun type* (β = -0.22, s.e. = 0.08, t = -2.66, p < 0.01, see Fig 2) showing that transportation scores were significantly higher when participants read stories with 1^st^ person pronouns. Again, EQ scores show an effect in the same direction as above (β = 0.03, s.e. = 0.01, t = 2.08, p < 0.05, see S1 Statistical Models *modelTRA*), meaning that a higher EQ predicts higher levels of transportation during reading.

The best model fit for **the emotional engagement subscale** data was produced when including both random intercepts and random slopes for participants and stories. *Pronoun type* shows no effect on the emotional engagement subscale (β = -0.11, s.e. = 0.17, t = -0.67, p = 0.50, see Fig 2). None of the individual differences measures contributed to the model (see S1 Statistical Models *modelEMO*).

The best model fit was produced when only including random intercepts for the **mental imagery subscale**. The model shows an effect of *pronoun type*, indicating that less mental imagery occurred in stories with 3^rd^ person pronouns compared to 1^st^ person pronoun stories (β = -0.21, s.e. = 0.10, t = -2.20, p < 0.05, S1 Statistical Models *modelIMA*, see Fig 2). None of the individual differences measures contributed to the model.

The best model fit was produced when only including random intercepts for the **narrative understanding subscale.** There was no effect of *pronoun type* (β = -0.09, s.e. = 0.10, t = -0.90, p = 0.37, see S1 Statistical Models *modelUND*, see Fig 2). None of the individual differences measures contributes to the model.

### Rating

The best model fit was produced when only including random intercepts for *Rating*. *Rating* is the only measure for which we observe an effect of text modification. Stories which were not modified for the experiment were rated better than stories which were modified (β = -0.33, s.e. = 0.15, t = -2.27, p < 0.05, see S1 Statistical Models *modelb3*). There was no effect of *pronoun type* (β = -0.18, s.e. = 0.14, t = -1.24, p = 0.22). In addition, we see that *Immersion* shows a highly significant effect as a predictor of *Rating* (β = 1.18, s.e. = 0.07, t = 16.87, p < 0.001, see S1 Statistical Models *modelALLrate*), indicating that higher degrees of immersion lead to higher rating scores. Finally, in the model including individual difference measures we see that ART shows an effect on *Rating* (β = 0.12, s.e. = 0.05, t = 2.37, p < 0.05; see S1 Statistical Models *modelFULLrate*), meaning that the ART score partly explains the rating data, whereby a higher ART score predicts higher rating.

### Ranking

**Fig 3:**
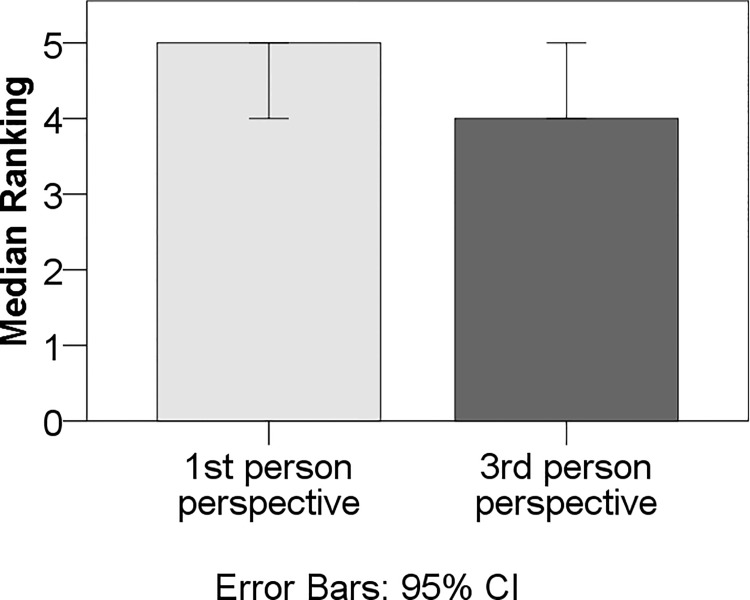
Effect of Pronoun type on ranking of the stories for appreciation. The effect of pronoun type on appreciation of stories as measured by ranking of all stories by how much participants liked them was statistically at p = 0.06. Note that Ranking is a non-normally distributed variable, so medians are plotted instead of means. Error bars represent 95% confidence intervals**. **

The best model fit was produced when only including random intercepts for *Ranking*. The effect of *pronoun type* for *Ranking* is significant at p = 0.06 (β = 0.39, s.e. = 0.21, t = -1.87, p = 0.06, see S1 Statistical Models *modelc3*, Fig 3). None of the individual difference measures contribute to the model (see S1 Statistical Models *modelFULLrank*). *Immersion* shows a highly significant effect as a predictor of *Ranking* (β = 0.59, s.e. = 0.14, t = 4.35, p< 0.001, see S1 Statistical Models *modelALLrank*).

### EDA

**Fig 4:**
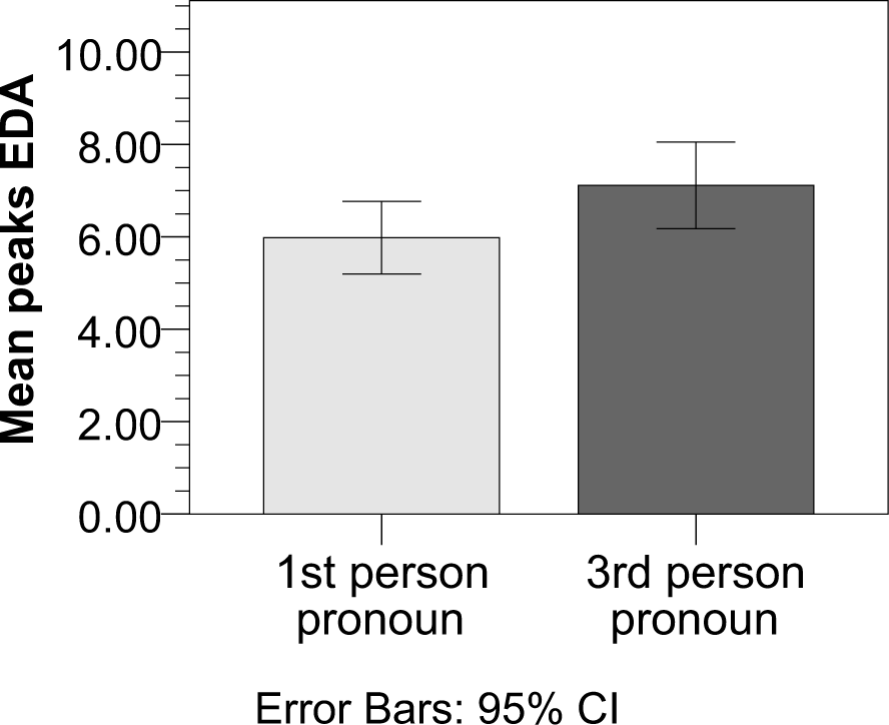
Peaks in EDA during reading stories with 1^st^ and 3^rd^ person pronouns referring to the main character. Number of peaks and valleys were computed using a peak detection algorithm in which peaks are defined as local maxima surrounded by valleys (d = 0.15). Number of peaks was significantly higher when participants read 3^rd^ person compared to 1^st^ person pronoun stories at p = 0.06. Error bars represent 95% confidence intervals.

The best model fit for the EDA data was produced when including both random intercepts and random slopes for participants and stories. *Pronoun type* shows an effect on the EDA measure meaning that stories with 3^rd^ person pronouns showed a higher number of peaks in the EDA signal compared to 1^st^ person pronoun stories, a difference which almost reached statistical significance (β = 1.04, s.e. = 0.55, t = 1.89, p = 0.06, see S1 Statistical Models *modeld2slopes*, Fig 4). None of the individual differences measures contributed to the effect (see S1 Statistical Models *modelEDA_FULL*), and neither did any of the other dependent variables show a significant link with the number of peaks in the EDA signal (see S1 Statistical Models *modelEDA_ALL*).

The control model with durations instead of peaks as dependent variable showed no effect of pronoun type (β = 1138, s.e. = 2191, t = 0.52, p = 0.60, see S1 Statistical Models *model_duration*).

## Discussion

The present study investigated the impact of personal pronouns referring to protagonists on readers’ engagement with literary stories. Participants read short stories from Dutch literature in which either 1^st^ or 3^rd^ person pronouns referred to the main character, whose viewpoint the story is narrated from. Electrodermal activity (EDA) was measured while participants read the stories. After reading each story, participants rated the story and filled out an immersion questionnaire. Finally, we asked participants to rank all stories for liking and collected several measures of inter-individual difference such as EQ score and prior print exposure.

The results show that stories with 1^st^ person pronouns lead to higher levels of overall immersion as measured by the questionnaire, which is in line with our predictions. We qualified this general difference by investigating the subscales of the immersion questionnaire. The effect of pronoun was present in the subscales *transportation* and *mental imagery*, again with 1^st^ person pronouns leading to higher scores. Additionally, we observed a relation between the scores on the immersion questionnaire and appreciation of a story. This shows that a story in which a participant scores high on immersion also receives a higher score in the appreciation rating and the story is more likely to be ranked high for appreciation. The relation between immersion and appreciation confirms the link between immersion and enjoyment of reading, as suggested earlier, e.g. [14,11]. In addition, our results suggest that people who score high on the EQ questionnaire also seem to get immersed more easily. Interestingly, the effect of our second major dependent measure, EDA during reading, showed an effect in the opposite direction. Here we observe more peaks in the EDA signal when participants read stories with 3^rd^ as compared to 1^st^ person pronouns, which is contrary to the direction of the effect in the scores of immersion and the appreciation measures. EDA is a measure for arousal, which can reflect emotional response, increased mental workload, and startle [43], thus there may be several reasons for observing more peaks in the EDA signal when participants read stories with 3^rd^ person pronouns. We want to point out that the direction of the effect in the EDA signal was not expected and that the following interpretation is post hoc. Moreover, the effect on EDA peaks was not large, and we interpret this finding with caution.

An obvious explanation is that the peaks in the EDA in fact reflect the level of immersion and emotional engagement (suspense) with the story and that the online measure of arousal is a better indicator of immersion. This would mean, however, that all behavioural measures used in this experiment are completely off. We consider this possibility unlikely given their status as standard measures [14,15] and the limited knowledge we have regarding EDA measures in experiments with longer trials.

Another possible explanation is related to embodied cognition accounts, according to which language is processed in 1^st^ person perspective by default, e.g. [56]. According to this view, linguistic input in 1^st^ person perspective like with 1^st^ person pronouns is already tailored to the cognitive system and promotes processing by decreasing mental workload. This means that language in 1^st^ person perspective can be processed directly by mapping information to the relevant modalities in way similar to a 1^st^ person experience. Language in 3^rd^ person perspective on the other hand requires additional processing before integration of information can take place. That means that 3^rd^ person linguistic input has to be ‘translated’ to fit a 1^st^ person experiencing system. Those additional processes could for example comprise a form of 'translation' of the information by transposition and mapping information to the reader’s perceptual system. Those additional processes require cognitive resources and effort, which can potentially be reflected in an effort effect in the EDA signal. This interpretation is supported by the fact that we do see the effect of the pronoun manipulation in the story- or plot-related components like *transportation* and *mental imagery* of the immersion questionnaire, while this was not the case for the directly related to the character *emotional engagement* component, which was not affected by choice of personal pronoun. This suggests that the effect we observe in the EDA is not related to 'social perspective taking' or emotional response, but rather showing an effect of decreased processing demands for 1^st^ person perspective. This interpretation supports accounts which claim that language in 1^st^ person perspective has processing benefits as compared to language in 3^rd^ person perspective, e. g. [9,56]. However, we want to be cautious with this interpretation as we do not observe an effect of pronoun type in the *understanding* and the *attention* components of the immersion questionnaire. This is likely due to the fact that the self-reported questionnaire taps into a different level of comprehension, but with the present data we are not able to distinguish clearly between different levels of comprehension.

Alternatively, the difficulty effect could also reflect natural perspective shifts, which are typical for narratives with internal focalization with 3^rd^ person pronouns referring to the character [57]. This means that perspective shifts in comprehension occur with several characters and not only with the protagonist. The reader steps in the shoes of the characters when trying to understand information about them, but otherwise processes the narrative from the perspective of an observer or another character. Those perspective shifts lead to increased processing cost. With 1^st^ person narration, the perspective of the narrator is identical to the protagonist from whose viewpoint the events are constructed, whereas with 3^rd^ person narrative and internal focalization, the viewpoint remains with the character, but now the story is presented by a (presumably) absent narrator. While it is intuitive that the 1^st^ person narrative has more "immediacy" and might promote identification (see also[58], the mechanism behind this is unclear.

Another potential explanation for the direction of the EDA effect relates to the scope of anticipation people do in language comprehension. While for a 1^st^ person perspective simulation it is only necessary to anticipate from the viewpoint of one character (and his or her understanding of other characters), an observer in the 3^rd^ person perspective is likely to anticipate from multiple viewpoints and potentially takes the perspective of multiple characters into account, keeping information from other characters active. This is clearly illustrated when we think about watching a horror movie: people are already excited or even scared before something is about to happen and feel the urge to warn the protagonist, e.g. since Alfred Hitchcock's famous shower scene in 'Psycho' we anticipate a terrible incident as soon as the camera is depicting a remarkably ordinary scene for just a bit too long. In such cases, the respective character however is not scared at all, because he or she only anticipates from his/her very own viewpoint. The reason for this is because we do not only take the perspective of the protagonist, but also the perspectives and motivations of other characters and the narrator (in this case the director) into account and make predictions based on our knowledge about the story (e.g. genre) or its characters. However, we know little about which type of information readers anticipate and if the perspective of multiple characters is taken into account. Future research is needed to test this objective. The last three potential explanations of the EDA effect are not mutually exclusive and it is likely that an interaction of all three causes leads to the observed effect. In contrast, the first explanation which argues for an effect of stronger immersion for 3^rd^ person stories is not compatible with the other three alternatives.

We have shown that personal pronouns can indeed be a crucial factor in how readers experience fiction. However, personal pronouns are only one possible facet of narrative viewpoint and narrative perspective. Whether the effects we observed can be generalized across several features of narrative style remains an open question (see [33], chapter 7 for discussion). Our results show that readers are more easily immersed when reading 1^st^ person stories, as proposed by narrative theory, e.g. [18]. We add to this assumption not only by providing experimental evidence, but also, we could show that the difference in processing 1^st^ or 3^rd^ person viewpoints in story engagement mainly relates to arousal and immersion, particularly transportation and experiencing mental imagery during reading. Further, our study adds to the field of discourse comprehension by showing that 3^rd^ person pronouns as discourse anchors seem to induce increased processing demands as compared to 1^st^ person pronouns, which in turn could account for lower immersion. This finding can be interpreted as evidence in support of embodied models of language processing. In addition, this study confirms the link between immersion and appreciation of the story and reveals evidence that appreciation of stories is positively linked to prior reading experience as measured by the ART. Moreover, our study confirms previous findings that individual differences in empathy skills (as measured by the EQ) are related to subjective experience during reading [46,47,49,50]. A remaining issue is whether pronouns are a major force in driving narrative perspective. It could be that people tend to identify with the character from whose viewpoint the story is told (see [25], which is independent of pronoun choice. As all stories we selected were internally focalized, the main character always told the story from his or her perspective. Another very plausible reason is variability between individuals. It has been shown that subjects differ substantially in perspective taking preferences [59]. Textual features such as personal pronouns are not always sufficient to overcome personal preferences [59]. Future research is needed to confirm our findings on other levels of discourse.

## Supporting Information

S1 Author Recognition Test (Dutch version)
(DOCX)

S1 Immersion Questionnaire (DOCX)

S1 Statistical Models (DOCX)

S1 Story Example A: Officina Asmara. (DOCX)

S2 Story Example B: The Mexican dog. (DOCX)

S3 Story Example C: River. (DOCX)

S1 Story Summaries and Content Questions (DOCX)

## References

[1] Gernsbacher MA. Two decades of structure building. Discourse Process. 1997;23: 265–304. 25484476 10.1080/01638539709544994 PMC4255941

[2] Zwaan RA, van Oostendorp H. Do readers construct spatial representations in naturalistic story comprehension? Discourse Process. 1993;16: 125–143. 10.1080/01638539309544832

[3] Csikszentmihalyi M. Flow : the psychology of optimal experience New York: Harper & Row; 1990.

[4] Sestir M, Green MC. You are who you watch: Identification and transportation effects on temporary self-concept. Soc Influ. 2010;5: 272–288. 10.1080/15534510.2010.490672

[5] Kuijpers MM. Absorbing Stories The effects of textual devices on absorption and evaluative responses. Utrecht; 2014.

[6] Jacobs AM. Neurocognitive poetics: methods and models for investigating the neuronal and cognitive-affective bases of literature reception. Front Hum Neurosci. 2015;9: 186 10.3389/fnhum.2015.00186 25932010 PMC4399337

[7] Jacobs AM. Towards a neurocognitive poetics model of literary reading In: Willems RM, editor. Cognitive Neuroscience of Natural Language Use. Cambridge University Press; 2015 pp. 135–159.

[8] Schrott R, Jacobs AM. Gehirn und Gedicht: Wie wir unsere Wirklichkeiten konstruieren (Brain and Poetry: How We Construct Our Realities) Munich: Hanser; 2011.

[9] Zwaan RA. The Immersed Experiencer: Toward an Embodied Theory of Language Comprehension. Psychol Learn Motiv Adv Res theory. 2004;44: 35–62.

[10] Gerrig RJ. Experiencing narrative worlds New Haven: Yale University Press; 1993.

[11] Green MC. Transportation Into Narrative Worlds: The Role of Prior Knowledge and Perceived Realism. Discourse Process. 2004;38: 247–266.

[12] Green MC, Brock TC. The Role of Transportation in the Persuasiveness of Public Narratives. J Pers Soc Psychol. 2000;79: 701–721. 10.1037//0022-3514.79.5.701 11079236

[13] Burke M. The rhetorical neuroscience of style: On the primacy of style elements during literary discourse processing. J Lit Semant. 2013;42: 199–215. 10.1515/jls-2013-0010

[14] Busselle R, Bilandzic H. Measuring Narrative Engagement. Media Psychol. 2009;12: 321–347. 10.1080/15213260903287259

[15] Kuijpers MM, Hakemulder F, Tan ES, Doicaru MM. Exploring absorbing reading experiences: Developing and validating a self-report scale to measure story world absorption. Sci Study Lit. 2014;4: 89–122. 10.1075/ssol.4.1.05kui

[16] Boyd B. Literature and Evolution: A Bio-Cultural Approach. Philos Lit. 2005;29: 1–23. 10.1353/phl.2005.0002

[17] Sanford AJ, Emmott C. Mind, Brain, Narrative Cambridge: Cambridge University Press; 2012.

[18] Oatley K. Meeting of Minds: Dialogue, Sympathy, and Identification in Reading Fiction. Poetics. 1999;26: 439–454.

[19] Albrecht JE, O’Brien EJ, Mason R a, Myers JL. The role of perspective in the accessibility of goals during reading. J Exp Psychol Learn Mem Cogn. 1995;21: 364–72. 7738505 10.1037//0278-7393.21.2.364

[20] Horton WS, Rapp DN. Out of sight, out of mind: occlusion and the accessibility of information in narrative comprehension. Psychon Bull Rev. 2003;10: 104–110. 12747496 10.3758/bf03196473

[21] de Graaf a., Hoeken H, Sanders J, Beentjes JWJ. Identification as a Mechanism of Narrative Persuasion. Communic Res. 2011;39: 802–823. 10.1177/0093650211408594

[22] Bal M. Narratology: Introduction to the Theory of Narrative. 2nd Editio. University of Toronto Press; 1997.

[23] Genette G. Narrative Discourse Cornell University Press; 1980.

[24] Rimmon-Kennan S. Narrative Fiction. 2nd Editio London & New York: Routledge; 2002.

[25] Bower GH, Morrow DG. Mental Models in Narrative Comprehension. Science. 1990;247: 4–8. 10.1126/science.2403694 2403694

[26] Johnson-Laird PN. Mental Models: Towards a Cognitive Science of Language, Inference and Consciousness Harvard University Press; 1983.

[27] Zacks JM, Michelon P. Transformations of Visuospatial Images. Behav Cogn Neurosci Rev. 2005;4. 10.1177/1534582305281085 16251727

[28] Kessler K, Thomson LA. The embodied nature of spatial perspective taking: embodied transformation versus sensorimotor interference. Cognition. Elsevier B.V.; 2010;114: 72–88. 10.1016/j.cognition.2009.08.015 19782971

[29] Frith CD, Frith U. Social cognition in humans. Curr Biol. 2007;17: R724–32. 10.1016/j.cub.2007.05.068 17714666

[30] Komeda H, Tsunemi K, Inohara K, Kusumi T, Rapp DN. Beyond disposition: the processing consequences of explicit and implicit invocations of empathy. Acta Psychol (Amst). Elsevier B.V.; 2013;142: 349–55. 10.1016/j.actpsy.2013.01.002 23422288

[31] Black J, Turner T, Bower G. Point of view in narrative comprehension, memory, and production. J Verbal Learn Verbal …. 1979;18: 187–198.

[32] Herman D. Story Logic Problems and Possibilities of Narrative. Lincoln, NE: University of Nebraska Press; 2002.

[33] Dancygier B. Conclusion: Multiple Viewpoints, Multiple Spaces Viewpoint in Language A Multimodal Perspective. Cambridge University Press; 2014 pp. 219–231.

[34] Bergen B, Chang N. Embodied Construction Grammar in Simulation-Based Language Understanding. In: Ostman J-O, Fried M, editors. Construction grammars: Cognitive grounding and theoretical extensions. Amsterdam; 2005. pp. 147–190.

[35] Brunyé TT, Ditman T, Mahoney CR, Augustyn JS, Taylor H a. When You and I share Perspectives: Pronouns modulate Perspective Taking during Narrative Comprehension. Psychol Sci. 2009;20: 27–32. 10.1111/j.1467-9280.2008.02249.x 19076318

[36] Brunyé TT, Ditman T, Mahoney CR, Taylor H a. Better you than I: Perspectives and emotion simulation during narrative comprehension. J Cogn Psychol. 2011;23: 659–666. 10.1080/20445911.2011.559160

[37] Ditman T, Brunyé TT, Mahoney CR, Taylor H a. Simulating an Enactment Effect: Pronouns guide Action Simulation during Narrative Comprehension. Cognition. Elsevier B.V.; 2010;115: 172–178. 10.1016/j.cognition.2009.10.014 19939357

[38] Papeo L, Corradi-dellʼacqua C, Rumiati RI. “She” is not like “I”: The tie between language and action is in our imagination “She” Is Not Like “I”: The Tie between Language and Action Is in Our Imagination. J Cogn Neurosci. 2011;23 10.1162/jocn 21671735

[39] Hétu S, Grégoire M, Saimpont A, Coll M-P, Eugène F, Michon P-E, et al The Neural Network of Motor Imagery: an ALE Meta-Analysis. Neurosci Biobehav Rev. Elsevier Ltd; 2013;37: 930–49. 10.1016/j.neubiorev.2013.03.017 23583615

[40] Ruby P, Decety J. Effect of Subjective Perspective Taking during Simulation of Action: a PET Investigation of Agency. Nat Neurosci. 2001;4: 546–50. 10.1038/87510 11319565

[41] de Hoop H, Tarenskeen S. It’s all about you in Dutch. J Pragmat. Elsevier B.V.; 2014; 10.1016/j.pragma.2014.07.001

[42] Boucsein W. Electrodermal Activity New York: Springer; 2012.

[43] Figner B, Murphy RO. Using skin conductance in judgment and decision making research In: Schulte-Mecklenbeck M, Kuehberger A, Ranyard R, editors. A Handbook of Process Tracing Methods for Decision Research. New York: NY: Psychology Press; 2011.

[44] Kreibig SD, Gendolla GHE. Autonomic Nervous System Measurement of Emotion in Education and Achievement Settings In: Pekrun R, Linnenbrink-Garcia L, editors. International Handbook of Emotions in Education. New York, London: Routledge; 2014 pp. 625–642.

[45] Bal PM, Veltkamp M. How does fiction reading influence empathy? An experimental investigation on the role of emotional transportation. PLoS One. 2013;8: e55341 10.1371/journal.pone.0055341 23383160 PMC3559433

[46] Keen S. Empathy and the Novel [Internet]. Oxford University Press; 2007 Available: http://books.google.com/books?hl=en&lr=&id=qdckVHP_nRAC&oi=fnd&pg=PR31&dq=Empathy+and+the+Novel&ots=WT5UiaTB2Z&sig=60XU3x4HlaV404uWkhOx6jFKtVM

[47] Kidd DC, Castano E. Reading Literary Fiction Improves Theory of Mind. Science (80-). 2013;342: 377–80. 10.1126/science.1239918 24091705

[48] Koopman EM (Emy). Empathic reactions after reading: The role of genre, personal factors and affective responses. Poetics. Elsevier B.V.; 2015;50: 62–79. 10.1016/j.poetic.2015.02.008

[49] Mar R a., Oatley K, Hirsh J, dela Paz J, Peterson JB. Bookworms versus Nerds: Exposure to Fiction versus Non-Fiction, Divergent Associations with Social Ability, and the Simulation of Fictional Social Worlds. J Res Pers. 2006;40: 694–712. 10.1016/j.jrp.2005.08.002

[50] Mar R a., Oatley K, Peterson JB. Exploring the Link between Reading Fiction and Empathy: Ruling out Individual Differences and examining Outcomes. Communications. 2009;34 10.1515/COMM.2009.025

[51] Acheson DJ, Wellu JB, MacDonald MC. New and updated tests of print exposure and reading abilities in college students. Behav Res Methods. 2008;40: 278–89. 18411551 10.3758/brm.40.1.278 PMC3022331

[52] Davis MH. A multidimensional approach to individual differences in empathy. JSAS Cat Sel Doc Psychol. 1980;10.

[53] Baron-Cohen S, Wheelwright S. The Empathy Quotient: an Investigation of Adults with Asperger Syndrome or High Functioning Autism, and Normal Sex Differences. J Autism Dev Disord. 2004;34: 163–75. Available: http://www.ncbi.nlm.nih.gov/pubmed/15162935 15162935 10.1023/b:jadd.0000022607.19833.00

[54] Pinheiro J, Bates D, DebRoy S, Sarkar D, (R CoreTeam). Linear and Nonlinear Mixed Effects Models. [Internet]. 2014. Available: http://cran.r-project.org/package=nlme

[55] Baayen RH, Davidson DJ, Bates DM. Mixed-effects modeling with crossed random effects for subjects and items. J Mem Lang. Elsevier Inc.; 2008;59: 390–412. 10.1016/j.jml.2007.12.005

[56] Wilson AD, Golonka S. Embodied Cognition is Not What you Think it is. Front Psychol. 2013;4: 58 10.3389/fpsyg.2013.00058 23408669 PMC3569617

[57] Miall DS, Kuiken D. Shifting Perspectives: Readers’ Feelings and Literary Response In: van Peer W, Chatman S, editors. Narrative Perspective: Cognition and Emotion. SUNY Press; 1998.

[58] van Krieken K, Sanders J, Hoeken H. Viewpoint representation in journalistic crime narratives: An analysis of grammatical roles and referential expressions. J Pragmat. Elsevier B.V.; 2014; 10.1016/j.pragma.2014.07.012

[59] Vukovic N, Williams JN. Individual differences in spatial cognition influence mental simulation of language. Cognition. 2015;142: 110–22. 10.1016/j.cognition.2015.05.017 26036923

