# Correction: Taking Perspective: Personal Pronouns Affect Experiential Aspects of Literary Reading

**DOI:** 10.1371/journal.pone.0157285

**Published:** 2016-06-03

**Authors:** The PLOS ONE staff

Fig 2 is incorrectly duplicated from Fig. 3. The authors have provided a corrected version here. The publisher apologizes for the error.

**Fig 2:**
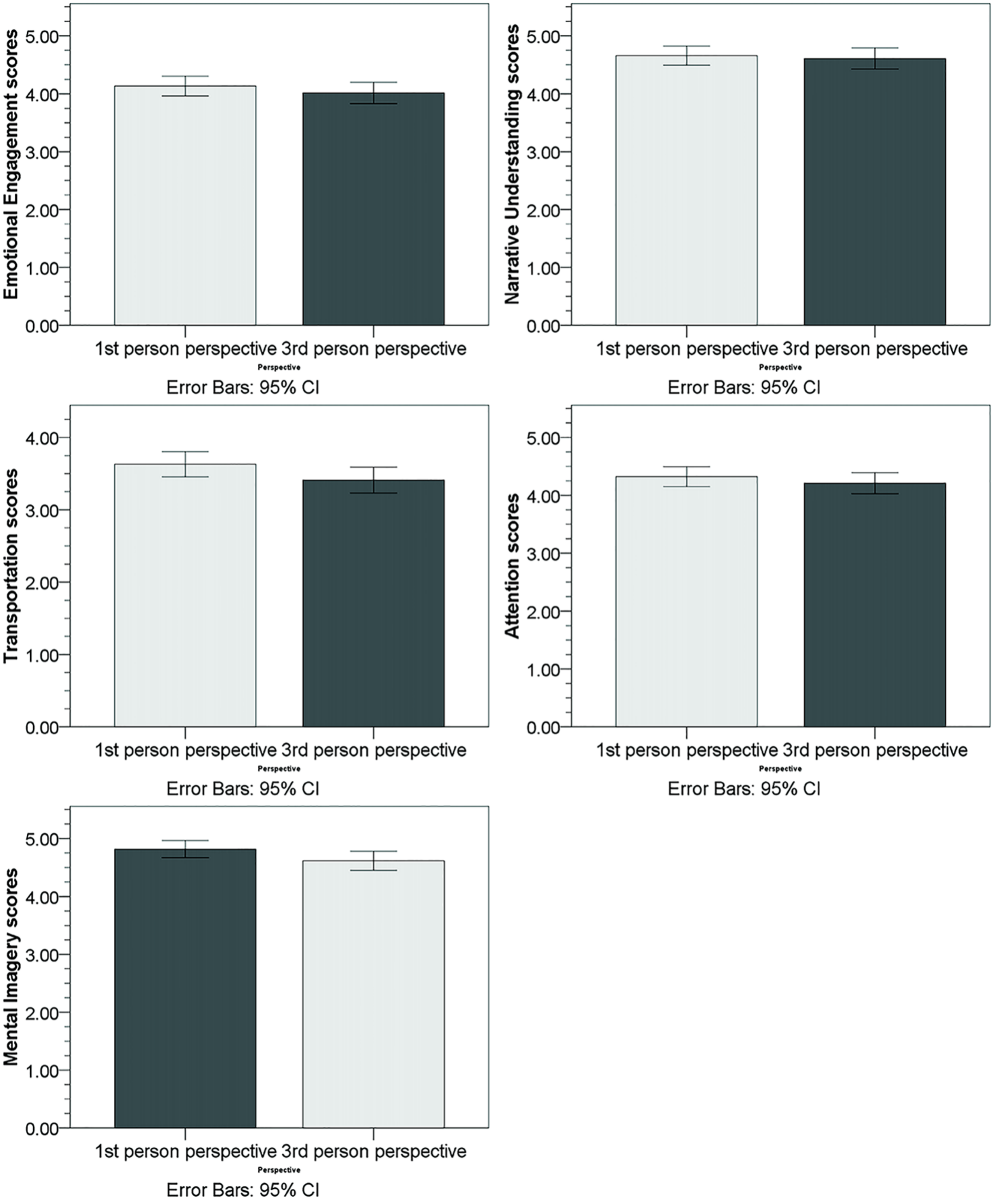
Subscales of the immersion questionnaire. The subscales were emotional engagement, narrative understanding, transportation, attention, and mental imagery. Differences between stories with 1st and 3rd person pronouns referring to the protagonist were significant for the transportation and the mental imagery subscale.
