# Supplementary material for: Taking Perspective: Personal Pronouns Affect Experiential Aspects of Literary Reading
Source: PLoS One. 2016 May 18;11(5):e0154732. doi: 10.1371/journal.pone.0154732 (PMC4883771; doi:10.1371/journal.pone.0154732)
Supplement: S1 Author Recognition Test — (Dutch version) (DOCX) [file pone.0154732.s001.docx]

# S1 Author Recognition Test (ART, Dutch version)

Below you see a list of names, some of which are names or pseudonyms of fiction authors, some are made up. Indicate by underlining, which names are familiar to you. When you underline a wrong name, it will be substracted from your final score. Therefore, please do not guess, but only underline names, you know for sure. It is not necessary to have read works from the writers though. (Fictive names marked here in italics).

Jonathan Franzen

*J.B. Guthrie*

Marek van der Jagt

Anna Blaman

Willem Kloos

Albert Camus

Mensje van Keulen

*Isabelle Liberman*

Robert Vuijsje

Jennifer Egan

*Gerald Duffy*

*Mark Sorenson*

Stephan Enter

René Appel

Tatiana de Rosnay

William Faulkner

Saskia Noort

Stephen King

Dimitri Verhulst

*H.P. Vliegenthart*

*Janet de Waal*

Dave Eggers

Herta Müller

*Robert Tierney*

John LeCarré

W.G. Sebald

Herman Koch

*Diane Corter*

Esther Verhoef

Italo Calvino

Toni Morrison

*Erik Bogaart*

Heleen van Royen

Jenna Blum

Douglas Adams

*Arnon Iffegem*

Isaac Asimov

Danielle Steel

*Sophie Boomgaarden*

*Andries Blok*

Terry Pratchett

John Grisham

**Categories:**

**Literature Dutch**

Marek van der Jagt

Anna Blaman

Willem Kloos

Mensje van Keulen

Stephan Enter

Dimitri Verhulst

**Literature international**

Jonathan Franzen

Dave Eggers

Herta Müller

Italo Calvino

W.G. Sebald

Toni Morrison

Amy Tan

Albert Camus

William Faulkner

**Fiction Dutch**

Saskia Noort

Heleen van Royen

Robert Vuijsje

Herman Koch

Esther Verhoef

René Appel

**Fiction international**

John LeCarré

Tatiana de Rosnay

Jenna Blum

Stephen King

Douglas Adams

Terry Pratchett

Isaac Asimov

Danielle Steel

John Grisham
