# Supplementary material for: Taking Perspective: Personal Pronouns Affect Experiential Aspects of Literary Reading
Source: PLoS One. 2016 May 18;11(5):e0154732. doi: 10.1371/journal.pone.0154732 (PMC4883771; doi:10.1371/journal.pone.0154732)
Supplement: S1 Immersion Questionnaire — (DOCX) [file pone.0154732.s002.docx]

# S1 Immersion questionnaire

**Instructions:** To what degree do the following statements correspond to your feelings and experiences while reading the story? Indicate with a cross on the scale which number is representative of how well the statement describes your experience (1=not at all, 7= completely).

Attention

1. While reading the story, I lost track of time.
2. I found it difficult to stay focused.
3. My attention was so focused on the story that I forgot about the surroundings.
4. At times, I completely forgot that I was in the middle of an experiment.
5. I was so concentrated on the reading that I forgot the world around me.
6. I was immersed in the story during reading.
7. I wanted to find out how the story ended.

Mental Imagery

1. While reading, I had an image of the main character in my mind.
2. While reading, I could see images of the situations being described.
3. At times, I could see the settings/environment in which the story unfolds in my mind.
4. At times, I had the feeling that I could see right through the eyes of the main character.

Emotional Engagement

1. I felt the same as the main character.
2. I shared the emotions of the main character.
3. I knew exactly what the characters were going through emotionally.
4. I never really felt like the main character felt.
5. The story affected me emotionally.
6. I could empathize with the characters.
7. I was able to understand the events in the story in a way similar to the way the characters understood them.
8. I could easily imagine myself in the situation of some of the characters.

Transportation

1. I forgot my own problems and concerns during the story.
2. When I finished reading the story, it felt like I had travelled into the world in which the story was set.
3. While reading, it seemed as if I was inside the narrative world.
4. While reading, my body was in the room, but my mind was inside the world created by the story.
5. At times, the world of the story and reality seemed to overlap.

Narrative Understanding

1. I could easily follow the thread of the story.
2. I understood why the events unfolded the way they did.
3. At certain points, I had a hard time making sense of what was going on in the story.
4. The story flows very well.
5. I understood why the characters did what they did.
6. I could understand why the characters felt the way they felt.
7. It was difficult to understand why the characters reacted to situations as they did.
