# Supplementary material for: Taking Perspective: Personal Pronouns Affect Experiential Aspects of Literary Reading
Source: PLoS One. 2016 May 18;11(5):e0154732. doi: 10.1371/journal.pone.0154732 (PMC4883771; doi:10.1371/journal.pone.0154732)
Supplement: S1 Statistical Models — (DOCX) [file pone.0154732.s003.docx]

## S1 Statistical models

For all full models including the individual differences only 50 subjects are included in the model, because 1 subject did not fill in the entire form of the EQ questionnaire and another subject selected all options for RH3. The abbreviations are the following:

ART: Author Recognition Test (measures print exposure)

EQ: Empathy Quotient

RH1: ‘Do you like fiction?’

RH2: ‘How often do you read?’

RH3: ‘How many books do you read per year?’

RH4: Number of liked fiction genres

FantasyScaleIRI: Fantasy Scale from the Interpersonal Reactivity Index

***Immersion***

> modela2 = lmer (Immersion ~ Perspective + Order + (1|Subject)+(1|Story), data=ndata)

> summary(modela2)

Linear mixed model fit by REML ['lmerMod']

Formula: Immersion ~ Perspective + Order + (1 | Subject) + (1 | Story)

Data: ndata

REML criterion at convergence: 1114.479

Random effects:

Groups Name Variance Std.Dev.

Subject (Intercept) 0.3095 0.5563

Story (Intercept) 0.2070 0.4549

Residual 0.6615 0.8133

Number of obs: 416, groups: Subject, 52; Story, 8

Fixed effects:

Estimate Std. Error t value

(Intercept) 4.15410 0.20628 20.138

Perspective2 -0.15822 0.07998 -1.978

Order2 0.33115 0.17369 1.907

Correlation of Fixed Effects:

(Intr) Prspc2

Perspectiv2 -0.194

Order2 -0.421 0.000

> anova (modela0, modela1, modela2, modela3)

Data: ndata

Models:

modela0: Immersion ~ 1 + (1 | Subject) + (1 | Story)

modela1: Immersion ~ Order + (1 | Subject) + (1 | Story)

modela2: Immersion ~ Perspective + Order + (1 | Subject) + (1 | Story)

modela3: Immersion ~ Perspective + Order + Manipulated + (1 | Subject) +

modela3: (1 | Story)

Df AIC BIC logLik deviance Chisq Chi Df Pr(>Chisq)

modela0 4 1123.5 1139.6 -557.73 1115.5

modela1 5 1121.9 1142.0 -555.93 1111.9 3.5965 1 0.05790 .

modela2 6 1120.0 1144.2 -553.99 1108.0 3.8897 1 0.04858 *

modela3 7 1121.8 1150.0 -553.88 1107.8 0.2244 1 0.63567

---

> PValues_Immersion <- Anova(modela2, type = 3, test = 'Chisq')

> PValues_Immersion

Analysis of Deviance Table (Type III Wald chisquare tests)

Response: Immersion

Chisq Df Pr(>Chisq)

(Intercept) 405.5276 1 < 2e-16 ***

Perspective 3.9135 1 0.04790 *

Order 3.6348 1 0.05658 .

---

Signif. codes: 0 ‘***’ 0.001 ‘**’ 0.01 ‘*’ 0.05 ‘.’ 0.1 ‘ ’ 1

> modelFULLimmersion= lmer (Immersion ~ Perspective + Order + ART + EQ + RH1 + RH2 + RH3 + RH4 + FantasyScaleIRI + (1|Subject) + (1|Story), data=ndata)

> summary (modelFULLimmersion)

Linear mixed model fit by REML ['lmerMod']

Formula: Immersion ~ Perspective + Order + ART + EQ + RH1 + RH2 + RH3 + RH4 + FantasyScaleIRI + (1 | Subject) + (1 | Story)

Data: ndata

REML criterion at convergence: 1092.906

Random effects:

Groups Name Variance Std.Dev.

Subject (Intercept) 0.2788 0.5280

Story (Intercept) 0.2087 0.4568

Residual 0.6668 0.8166

Number of obs: 400, groups: Subject, 50; Story, 8

Fixed effects:

Estimate Std. Error t value

(Intercept) 3.912055 0.959444 4.077

Perspective2 -0.144724 0.081782 -1.770

Order2 0.249558 0.192572 1.296

ART 0.029525 0.028044 1.053

EQ 0.021726 0.008446 2.572

RH1 -0.118955 0.145316 -0.819

RH2 -0.014247 0.131029 -0.109

RH3 0.077816 0.072526 1.073

RH4 0.030852 0.049018 0.629

FantasyScaleIRI -0.139295 0.139315 -1.000

Correlation of Fixed Effects:

(Intr) Prspc2 Order2 ART EQ RH1 RH2 RH3 RH4

Perspectiv2 -0.043

Order2 -0.312 0.000

ART -0.473 0.000 0.176

EQ -0.086 0.000 -0.311 -0.048

RH1 -0.548 0.000 0.168 0.152 -0.007

RH2 0.008 0.000 -0.003 0.256 -0.115 -0.688

RH3 -0.092 0.000 0.294 0.007 -0.164 0.100 -0.193

RH4 -0.076 0.000 -0.009 -0.226 0.218 0.126 -0.302 0.178

FntsySclIRI -0.739 0.000 0.254 0.264 -0.334 0.310 0.102 -0.047 -0.321

> PValues_Immersion <- Anova(modelaFULL, type = 3, test = 'Chisq')

> PValues_Immersion

Analysis of Deviance Table (Type III Wald chisquare tests)

Response: Immersion

Chisq Df Pr(>Chisq)

(Intercept) 19.4048 1 1.057e-05 ***

Perspective 3.1271 1 0.07700 .

Order 1.4640 1 0.22630

Manipulated 0.3637 1 0.54647

Gender 2.2234 1 0.13594

ART 0.7939 1 0.37292

EQ 4.1980 1 0.04047 *

RH1 1.2564 1 0.26233

RH2 0.0002 1 0.98776

RH3 1.8681 1 0.17169

RH4 0.6973 1 0.40368

FantasyScaleIRI 1.2407 1 0.26533

---

Signif. codes: 0 ‘***’ 0.001 ‘**’ 0.01 ‘*’ 0.05 ‘.’ 0.1 ‘ ’ 1

Attention Subscale

> modelATT = lmer (Attention ~ Perspective + Order + Gender + ART + EQ + RH1 + RH2 + RH3 + RH4 + FantasyScaleIRI + (1|Subject)+(1|Story), data=ndata)

> summary(modelATT)

Linear mixed model fit by REML ['lmerMod']

Formula: Attention ~ Perspective + Order + Gender + ART + EQ + RH1 + RH2 + RH3 + RH4 + FantasyScaleIRI + (1 | Subject) + (1 | Story)

Data: ndata

REML criterion at convergence: 1223.214

Random effects:

Groups Name Variance Std.Dev.

Subject (Intercept) 0.4570 0.6760

Story (Intercept) 0.2020 0.4495

Residual 0.9241 0.9613

Number of obs: 400, groups: Subject, 50; Story, 8

Fixed effects:

Estimate Std. Error t value

(Intercept) 4.846253 1.246089 3.889

Perspective2 -0.139766 0.096272 -1.452

Order2 0.201727 0.242782 0.831

Gender2 -0.396865 0.244140 -1.626

ART -0.004291 0.035491 -0.121

EQ 0.022565 0.011121 2.029

RH1 -0.281196 0.186570 -1.507

RH2 -0.010580 0.165250 -0.064

RH3 0.154852 0.093031 1.665

RH4 0.075942 0.062170 1.222

FantasyScaleIRI -0.222683 0.175585 -1.268

Correlation of Fixed Effects:

(Intr) Prspc2 Order2 Gendr2 ART EQ RH1 RH2 RH3 RH4

Perspectiv2 -0.039

Order2 -0.321 0.000

Gender2 -0.271 0.000 0.074

ART -0.485 0.000 0.182 0.114

EQ -0.160 0.000 -0.274 0.297 -0.012

RH1 -0.575 0.000 0.179 0.202 0.171 0.054

RH2 0.029 0.000 -0.009 -0.078 0.245 -0.133 -0.687

RH3 -0.034 0.000 0.273 -0.198 -0.016 -0.212 0.056 -0.173

RH4 -0.037 0.000 -0.019 -0.131 -0.237 0.167 0.096 -0.288 0.199

FntsySclIRI -0.732 0.000 0.258 0.069 0.270 -0.297 0.317 0.096 -0.060 -0.327

> PValues_ATT <- Anova(modelATT, type = 3, test = 'Chisq')

> PValues_ATT

Analysis of Deviance Table (Type III Wald chisquare tests)

Response: Attention

Chisq Df Pr(>Chisq)

(Intercept) 15.4953 1 8.271e-05 ***

Perspective 2.1136 1 0.14599

Order 0.7192 1 0.39641

Manipulated 1.6365 1 0.20081

Gender 2.6353 1 0.10451

ART 0.0150 1 0.90240

EQ 4.1256 1 0.04224 *

RH1 2.3099 1 0.12855

RH2 0.0022 1 0.96251

RH3 2.8044 1 0.09400 .

RH4 1.5248 1 0.21689

FantasyScaleIRI 1.6049 1 0.20521

---

Signif. codes: 0 ‘***’ 0.001 ‘**’ 0.01 ‘*’ 0.05 ‘.’ 0.1 ‘ ’ 1

Transportation Subscale

> modelTRA = lmer (Transportation ~ Perspective + Order + Gender + ART + EQ + RH1 + RH2 + RH3 + RH4 + FantasyScaleIRI + (1|Subject)+(1|Story), data=ndata)

> summary(modelTRA)

Linear mixed model fit by REML ['lmerMod']

Formula: Transportation ~ Perspective + Order + Gender + ART + EQ + RH1 + RH2 + RH3 + RH4 + FantasyScaleIRI + (1 | Subject) + (1 | Story)

Data: ndata

REML criterion at convergence: 1107.638

Random effects:

Groups Name Variance Std.Dev.

Subject (Intercept) 0.81914 0.9051

Story (Intercept) 0.08012 0.2831

Residual 0.67903 0.8240

Number of obs: 392, groups: Subject, 49; Story, 8

Fixed effects:

Estimate Std. Error t value

(Intercept) 2.73010 1.62677 1.678

Perspective2 -0.22209 0.08336 -2.664

Order2 0.43684 0.30777 1.419

Gender2 -0.23945 0.30794 -0.778

ART 0.03118 0.04552 0.685

EQ 0.02900 0.01398 2.075

RH1 -0.08370 0.23757 -0.352

RH2 -0.06557 0.20789 -0.315

RH3 0.12576 0.12408 1.014

RH4 0.07489 0.07812 0.959

FantasyScaleIRI -0.16017 0.23417 -0.684

Correlation of Fixed Effects:

(Intr) Prspc2 Order2 Gendr2 ART EQ RH1 RH2 RH3 RH4

Perspectiv2 -0.026

Order2 -0.345 0.000

Gender2 -0.234 0.000 0.061

ART -0.516 0.000 0.204 0.094

EQ -0.146 0.000 -0.275 0.298 -0.017

RH1 -0.593 0.000 0.196 0.185 0.198 0.049

RH2 0.042 0.000 -0.015 -0.073 0.230 -0.131 -0.685

RH3 0.067 0.000 0.210 -0.157 -0.082 -0.191 -0.002 -0.146

RH4 -0.033 0.000 -0.020 -0.130 -0.234 0.168 0.093 -0.287 0.191

FntsySclIRI -0.762 0.000 0.285 0.036 0.316 -0.289 0.349 0.074 -0.166 -0.311

> PValues_TRA <- Anova(modelTRA, type = 3, test = 'Chisq')

> PValues_TRA

Analysis of Deviance Table (Type III Wald chisquare tests)

Response: Transportation

Chisq Df Pr(>Chisq)

(Intercept) 2.8044 1 0.094008 .

Perspective 7.0763 1 0.007811 **

Order 2.0108 1 0.156187

Manipulated 0.0227 1 0.880179

Gender 0.6053 1 0.436552

ART 0.4693 1 0.493296

EQ 4.3038 1 0.038027 *

RH1 0.1236 1 0.725149

RH2 0.1003 1 0.751428

RH3 1.0262 1 0.311060

RH4 0.9173 1 0.338194

FantasyScaleIRI 0.4682 1 0.493798

---

Signif. codes: 0 ‘***’ 0.001 ‘**’ 0.01 ‘*’ 0.05 ‘.’ 0.1 ‘ ’ 1

Emotional Engagement Subscale

> modelEMO = lmer (EmotionalEngagement ~ Perspective + Order + Gender + ART + EQ + RH1 + RH2 + RH3 + RH4 + FantasyScaleIRI + (1|Subject)+(1|Story), data=ndata)

> summary(modelEMO)

Linear mixed model fit by REML ['lmerMod']

Formula: EmotionalEngagement ~ Perspective + Order + Gender + ART + EQ + RH1 + RH2 + RH3 + RH4 + FantasyScaleIRI + (1 | Subject) + (1 | Story)

Data: ndata

REML criterion at convergence: 1278.737

Random effects:

Groups Name Variance Std.Dev.

Subject (Intercept) 0.2982 0.5460

Story (Intercept) 0.2757 0.5251

Residual 1.1149 1.0559

Number of obs: 400, groups: Subject, 50; Story, 8

Fixed effects:

Estimate Std. Error t value

(Intercept) 3.498874 1.096684 3.190

Perspective2 -0.119468 0.105743 -1.130

Order2 0.304651 0.212230 1.435

Gender2 -0.221903 0.213417 -1.040

ART 0.040158 0.031025 1.294

EQ 0.013494 0.009721 1.388

RH1 -0.107556 0.163091 -0.659

RH2 0.073136 0.144454 0.506

RH3 0.065434 0.081323 0.805

RH4 -0.017906 0.054346 -0.329

FantasyScaleIRI -0.016865 0.153489 -0.110

Correlation of Fixed Effects:

(Intr) Prspc2 Order2 Gendr2 ART EQ RH1 RH2 RH3 RH4

Perspectiv2 -0.048

Order2 -0.319 0.000

Gender2 -0.269 0.000 0.074

ART -0.482 0.000 0.182 0.114

EQ -0.159 0.000 -0.274 0.297 -0.012

RH1 -0.571 0.000 0.179 0.202 0.171 0.054

RH2 0.029 0.000 -0.009 -0.078 0.245 -0.133 -0.687

RH3 -0.034 0.000 0.273 -0.198 -0.016 -0.212 0.056 -0.173

RH4 -0.037 0.000 -0.019 -0.131 -0.237 0.167 0.096 -0.288 0.199

FntsySclIRI -0.727 0.000 0.258 0.069 0.270 -0.297 0.317 0.096 -0.060 -0.327

> PValues_EMO <- Anova(modelEMOslopes, type = 3, test = 'Chisq')

> PValues_EMO

Analysis of Deviance Table (Type III Wald chisquare tests)

Response: EmotionalEngagement

Chisq Df Pr(>Chisq)

(Intercept) 11.1511 1 0.0008398 ***

Perspective 0.4567 1 0.4991715

Order 2.3459 1 0.1256110

Manipulated 0.0098 1 0.9211202

Gender 1.5754 1 0.2094192

ART 1.7524 1 0.1855745

EQ 1.8372 1 0.1752784

RH1 0.4367 1 0.5087330

RH2 0.2998 1 0.5839841

RH3 0.3172 1 0.5732745

RH4 0.1175 1 0.7317332

FantasyScaleIRI 0.0059 1 0.9387692

---

Signif. codes: 0 ‘***’ 0.001 ‘**’ 0.01 ‘*’ 0.05 ‘.’ 0.1 ‘ ’ 1

Mental Imagery Subscale

> modelIMA= lmer (MentalImagery ~ Perspective + Order + Gender + ART + EQ + RH1 + RH2 + RH3 + RH4 + FantasyScaleIRI + (1|Subject)+(1|Story), data=ndata)

> summary(modelIMA)

Linear mixed model fit by REML ['lmerMod']

Formula: MentalImagery ~ Perspective + Order + Gender + ART + EQ + RH1 + RH2 + RH3 + RH4 + FantasyScaleIRI + (1 | Subject) + (1 | Story)

Data: ndata

REML criterion at convergence: 1168.497

Random effects:

Groups Name Variance Std.Dev.

Subject (Intercept) 0.32219 0.5676

Story (Intercept) 0.04252 0.2062

Residual 0.89502 0.9461

Number of obs: 392, groups: Subject, 49; Story, 8

Fixed effects:

Estimate Std. Error t value

(Intercept) 5.610666 1.128107 4.974

Perspective2 -0.210164 0.095678 -2.197

Order2 0.211202 0.213263 0.990

Gender2 -0.398713 0.213382 -1.869

ART 0.019231 0.031542 0.610

EQ 0.015810 0.009686 1.632

RH1 -0.247459 0.164618 -1.503

RH2 0.005097 0.144051 0.035

RH3 0.057765 0.085980 0.672

RH4 0.034882 0.054135 0.644

FantasyScaleIRI -0.212939 0.162266 -1.312

Correlation of Fixed Effects:

(Intr) Prspc2 Order2 Gendr2 ART EQ RH1 RH2 RH3 RH4

Perspectiv2 -0.042

Order2 -0.345 0.000

Gender2 -0.234 0.000 0.061

ART -0.516 0.000 0.204 0.094

EQ -0.146 0.000 -0.275 0.298 -0.017

RH1 -0.593 0.000 0.196 0.185 0.198 0.049

RH2 0.042 0.000 -0.015 -0.073 0.230 -0.131 -0.685

RH3 0.067 0.000 0.210 -0.157 -0.082 -0.191 -0.002 -0.146

RH4 -0.033 0.000 -0.020 -0.130 -0.234 0.168 0.093 -0.287 0.191

FntsySclIRI -0.761 0.000 0.285 0.036 0.316 -0.289 0.349 0.074 -0.166 -0.311

> PValues_IMA <- Anova(modelIMA, type = 3, test = 'Chisq')

> PValues_IMA

Analysis of Deviance Table (Type III Wald chisquare tests)

Response: MentalImagery

Chisq Df Pr(>Chisq)

(Intercept) 24.6908 1 6.731e-07 ***

Perspective 4.8103 1 0.02829 *

Order 0.9794 1 0.32236

Manipulated 0.0021 1 0.96327

Gender 3.4923 1 0.06166 .

ART 0.3718 1 0.54203

EQ 2.6645 1 0.10261

RH1 2.2584 1 0.13289

RH2 0.0012 1 0.97234

RH3 0.4510 1 0.50185

RH4 0.4146 1 0.51964

FantasyScaleIRI 1.7225 1 0.18937

---

Signif. codes: 0 ‘***’ 0.001 ‘**’ 0.01 ‘*’ 0.05 ‘.’ 0.1 ‘ ’ 1

Narrative Understanding Subscale

> modelUND = lmer (NarrativeUnderstanding ~ Perspective + Order + Gender + ART + EQ + RH1 + RH2 + RH3 + RH4 + FantasyScaleIRI + (1|Subject)+(1|Story), data=ndata)

> summary(modelUND)

Linear mixed model fit by REML ['lmerMod']

Formula: NarrativeUnderstanding ~ Perspective + Order + Gender + ART + EQ + RH1 + RH2 + RH3 + RH4 + FantasyScaleIRI + (1 | Subject) + (1 | Story)

Data: ndata

REML criterion at convergence: 1224.243

Random effects:

Groups Name Variance Std.Dev.

Subject (Intercept) 0.2094 0.4576

Story (Intercept) 0.5242 0.7240

Residual 0.9703 0.9850

Number of obs: 400, groups: Subject, 50; Story, 8

Fixed effects:

Estimate Std. Error t value

(Intercept) 4.673948 0.974023 4.799

Perspective2 -0.088275 0.098655 -0.895

Order2 0.065685 0.184497 0.356

Gender2 -0.236758 0.185528 -1.276

ART 0.042803 0.026970 1.587

EQ 0.010992 0.008451 1.301

RH1 -0.086510 0.141779 -0.610

RH2 -0.033210 0.125577 -0.264

RH3 0.052637 0.070696 0.745

RH4 0.049292 0.047244 1.043

FantasyScaleIRI -0.135247 0.133432 -1.014

Correlation of Fixed Effects:

(Intr) Prspc2 Order2 Gendr2 ART EQ RH1 RH2 RH3 RH4

Perspectiv2 -0.051

Order2 -0.312 0.000

Gender2 -0.263 0.000 0.074

ART -0.472 0.000 0.182 0.114

EQ -0.156 0.000 -0.274 0.297 -0.012

RH1 -0.559 0.000 0.179 0.202 0.171 0.054

RH2 0.028 0.000 -0.009 -0.078 0.245 -0.133 -0.687

RH3 -0.033 0.000 0.273 -0.198 -0.016 -0.212 0.056 -0.173

RH4 -0.036 0.000 -0.019 -0.131 -0.237 0.167 0.096 -0.288 0.199

FntsySclIRI -0.712 0.000 0.258 0.069 0.270 -0.297 0.317 0.096 -0.060 -0.327

> PValues_UND <- Anova(modelUND, type = 3, test = 'Chisq')

> PValues_UND

Analysis of Deviance Table (Type III Wald chisquare tests)

Response: NarrativeUnderstanding

Chisq Df Pr(>Chisq)

(Intercept) 23.3717 1 1.335e-06 ***

Perspective 0.8020 1 0.3705

Order 0.1382 1 0.7101

Manipulated 0.9870 1 0.3205

Gender 1.6117 1 0.2043

ART 2.5135 1 0.1129

EQ 1.6835 1 0.1945

RH1 0.3837 1 0.5356

RH2 0.0607 1 0.8053

RH3 0.5637 1 0.4528

RH4 1.1109 1 0.2919

FantasyScaleIRI 1.0177 1 0.3131

---

Signif. codes: 0 ‘***’ 0.001 ‘**’ 0.01 ‘*’ 0.05 ‘.’ 0.1 ‘ ’ 1

***Rating***

> modelb3 = lmer (Rating ~ Perspective + Order + Manipulated + (1|Subject)+(1|Story), data=ndata)

> summary(modelb3)

Linear mixed model fit by REML ['lmerMod']

Formula: Rating ~ Perspective + Order + Manipulated + (1 | Subject) + (1 | Story)

Data: ndata

REML criterion at convergence: 1562.872

Random effects:

Groups Name Variance Std.Dev.

Subject (Intercept) 0.9759 0.9879

Story (Intercept) 0.6239 0.7899

Residual 2.1075 1.4517

Number of obs: 408, groups: Subject, 51; Story, 8

Fixed effects:

Estimate Std. Error t value

(Intercept) 6.3427 0.3710 17.095

Perspective2 -0.1786 0.1442 -1.239

Order2 0.5029 0.3119 1.613

Manipulated2 -0.3301 0.1453 -2.272

Correlation of Fixed Effects:

(Intr) Prspc2 Order2

Perspectiv2 -0.195

Order2 -0.427 0.000

Manipulatd2 -0.189 0.004 -0.009

> anova (modelb0, modelb1, modelb2, modelb3)

Data: ndata

Models:

modelb0: Rating ~ 1 + (1 | Subject) + (1 | Story)

modelb1: Rating ~ Order + (1 | Subject) + (1 | Story)

modelb2: Rating ~ Perspective + Order + (1 | Subject) + (1 | Story)

modelb3: Rating ~ Perspective + Order + Manipulated + (1 | Subject) +

modelb3: (1 | Story)

Df AIC BIC logLik deviance Chisq Chi Df Pr(>Chisq)

modelb0 4 1575.0 1591.0 -783.49 1567.0

modelb1 5 1574.5 1594.5 -782.23 1564.5 2.5180 1 0.11255

modelb2 6 1575.0 1599.0 -781.49 1563.0 1.4868 1 0.22272

modelb3 7 1571.8 1599.9 -778.91 1557.8 5.1559 1 0.02317 *

---

> PValues_Rating <- Anova(modelb3, type = 3, test = 'Chisq')

> PValues_Rating

Analysis of Deviance Table (Type III Wald chisquare tests)

Response: Rating

Chisq Df Pr(>Chisq)

(Intercept) 292.2342 1 < 2e-16 ***

Perspective 1.5346 1 0.21543

Order 2.6011 1 0.10679

Manipulated 5.1619 1 0.02309 *

---

Signif. codes: 0 ‘***’ 0.001 ‘**’ 0.01 ‘*’ 0.05 ‘.’ 0.1 ‘ ’ 1

> modelFULLrate= lmer (Rating ~ Perspective + Order + ART + EQ + RH1 + RH2 + RH3 + RH4 + FantasyScaleIRI + (1|Subject) + (1|Story), data=ndata)

> summary (modelFULLrate)

Linear mixed model fit by REML ['lmerMod']

Formula: Rating ~ Perspective + Order + ART + EQ + RH1 + RH2 + RH3 + RH4 + FantasyScaleIRI + (1 | Subject) + (1 | Story)

Data: ndata

REML criterion at convergence: 1521.373

Random effects:

Groups Name Variance Std.Dev.

Subject (Intercept) 0.8892 0.9430

Story (Intercept) 0.6059 0.7784

Residual 2.1718 1.4737

Number of obs: 392, groups: Subject, 49; Story, 8

Fixed effects:

Estimate Std. Error t value

(Intercept) 4.45907 1.73209 2.574

Perspective2 -0.18173 0.14907 -1.219

Order2 0.44260 0.34910 1.268

ART 0.11983 0.05050 2.373

EQ 0.02114 0.01516 1.394

RH1 -0.09663 0.26035 -0.371

RH2 0.01839 0.23624 0.078

RH3 0.01773 0.13010 0.136

RH4 0.03525 0.08775 0.402

FantasyScaleIRI 0.03278 0.24941 0.131

Correlation of Fixed Effects:

(Intr) Prspc2 Order2 ART EQ RH1 RH2 RH3 RH4

Perspectiv2 -0.043

Order2 -0.328 0.000

ART -0.481 0.000 0.189

EQ -0.096 0.000 -0.294 -0.040

RH1 -0.549 0.000 0.172 0.156 -0.004

RH2 -0.009 0.000 0.016 0.266 -0.105 -0.678

RH3 -0.100 0.000 0.299 0.014 -0.158 0.102 -0.183

RH4 -0.074 0.000 -0.010 -0.225 0.217 0.126 -0.301 0.177

FntsySclIRI -0.731 0.000 0.250 0.262 -0.333 0.310 0.101 -0.048 -0.321

> PValues_FULLrate <- Anova(modelFULLrate, type = 3, test = 'Chisq')

> PValues_FULLrate

Analysis of Deviance Table (Type III Wald chisquare tests)

Response: Rating

Chisq Df Pr(>Chisq)

(Intercept) 6.6275 1 0.01004 *

Perspective 1.4862 1 0.22280

Order 1.6074 1 0.20485

ART 5.6311 1 0.01765 *

EQ 1.9443 1 0.16320

RH1 0.1378 1 0.71051

RH2 0.0061 1 0.93796

RH3 0.0186 1 0.89157

RH4 0.1613 1 0.68795

FantasyScaleIRI 0.0173 1 0.89542

---

Signif. codes: 0 ‘***’ 0.001 ‘**’ 0.01 ‘*’ 0.05 ‘.’ 0.1 ‘ ’ 1

> modelALLrate= lmer ( Rating ~ Perspective + Order + EQ + ART + Ranking + Immersion + PeaksD0.15 +(1|Subject)+(1|Story), data=ndata)

> summary(modelALLrate)

Linear mixed model fit by REML ['lmerMod']

Formula: Rating ~ Perspective + Order + EQ + ART + Ranking + Immersion + PeaksD0.15 + (1 | Subject) + (1 | Story)

Data: ndata

REML criterion at convergence: 1173.842

Random effects:

Groups Name Variance Std.Dev.

Subject (Intercept) 0.66594 0.8161

Story (Intercept) 0.03668 0.1915

Residual 0.90307 0.9503

Number of obs: 382, groups: Subject, 50; Story, 8

Fixed effects:

Estimate Std. Error t value

(Intercept) 0.319728 0.575482 0.556

Perspective2 0.071271 0.099147 0.719

Order2 0.097274 0.255292 0.381

EQ -0.003532 0.011291 -0.313

ART 0.072524 0.030311 2.393

Ranking 0.125301 0.027202 4.606

Immersion 1.181214 0.070032 16.867

PeaksD0.15 0.004112 0.010994 0.374

Correlation of Fixed Effects:

(Intr) Prspc2 Order2 EQ ART Rankng Immrsn

Perspectiv2 -0.103

Order2 -0.063 -0.001

EQ -0.767 -0.010 -0.162

ART -0.464 -0.003 -0.027 0.208

Ranking -0.019 0.045 0.029 0.067 0.045

Immersion -0.252 0.060 -0.066 -0.138 -0.097 -0.532

PeaksD0.15 -0.061 -0.124 -0.013 -0.069 -0.040 0.002 0.039

> PValues_rating <- Anova(modelALLrate, type = 3, test = 'Chisq')

> PValues_rating

Analysis of Deviance Table (Type III Wald chisquare tests)

Response: Rating

Chisq Df Pr(>Chisq)

(Intercept) 0.3087 1 0.57850

Perspective 0.5167 1 0.47223

Order 0.1452 1 0.70318

EQ 0.0979 1 0.75439

ART 5.7247 1 0.01673 *

Ranking 21.2180 1 4.099e-06 ***

Immersion 284.4843 1 < 2.2e-16 ***

PeaksD0.15 0.1399 1 0.70838

---

Signif. codes: 0 ‘***’ 0.001 ‘**’ 0.01 ‘*’ 0.05 ‘.’ 0.1 ‘ ’ 1

***Ranking***

> modelc2 = lmer (Ranking ~ Perspective + Order +(1|Subject)+(1|Story), data=ndata)

> summary(modelc2)

Linear mixed model fit by REML ['lmerMod']

Formula: Ranking ~ Perspective + Order + (1 | Subject) + (1 | Story)

Data: ndata

REML criterion at convergence: 1817.333

Random effects:

Groups Name Variance Std.Dev.

Subject (Intercept) 0.0000 0.0000

Story (Intercept) 0.9457 0.9725

Residual 4.4062 2.0991

Number of obs: 416, groups: Subject, 52; Story, 8

Fixed effects:

Estimate Std. Error t value

(Intercept) 4.68343 0.38736 12.091

Perspective2 -0.38610 0.20640 -1.871

Order2 0.01923 0.20583 0.093

Correlation of Fixed Effects:

(Intr) Prspc2

Perspectiv2 -0.266

Order2 -0.266 0.000

> anova (modelc0, modelc1, modelc2, modelc3)

Data: ndata

Models:

modelc0: Ranking ~ 1 + (1 | Subject) + (1 | Story)

modelc1: Ranking ~ Order + (1 | Subject) + (1 | Story)

modelc2: Ranking ~ Perspective + Order + (1 | Subject) + (1 | Story)

modelc3: Ranking ~ Perspective + Order + Manipulated + (1 | Subject) +

modelc3: (1 | Story)

Df AIC BIC logLik deviance Chisq Chi Df Pr(>Chisq)

modelc0 4 1825.9 1842.0 -908.95 1817.9

modelc1 5 1827.9 1848.0 -908.95 1817.9 0.0087 1 0.92571

modelc2 6 1826.4 1850.6 -907.20 1814.4 3.4844 1 0.06195 .

modelc3 7 1827.9 1856.1 -906.95 1813.9 0.5038 1 0.47785

> PValues_Ranking <- Anova(modelc2, type = 3, test = 'Chisq')

> PValues_Ranking

Analysis of Deviance Table (Type III Wald chisquare tests)

Response: Ranking

Chisq Df Pr(>Chisq)

(Intercept) 146.1868 1 < 2e-16 ***

Perspective 3.4994 1 0.06139 .

Order 0.0087 1 0.92556

---

Signif. codes: 0 ‘***’ 0.001 ‘**’ 0.01 ‘*’ 0.05 ‘.’ 0.1 ‘ ’ 1

> modelFULLrank= lmer (Ranking ~ Perspective + Order + ART + EQ + RH1 + RH2 + RH3 + RH4 + FantasyScaleIRI + (1|Subject) + (1|Story), data=ndata)

> summary (modelFULLrank)

Linear mixed model fit by REML ['lmerMod']

Formula: Ranking ~ Perspective + Order + ART + EQ + RH1 + RH2 + RH3 + RH4 + FantasyScaleIRI + (1 | Subject) + (1 | Story)

Data: ndata

REML criterion at convergence: 1774.419

Random effects:

Groups Name Variance Std.Dev.

Subject (Intercept) 0.0000 0.0000

Story (Intercept) 0.9276 0.9631

Residual 4.4979 2.1208

Number of obs: 400, groups: Subject, 50; Story, 8

Fixed effects:

Estimate Std. Error t value

(Intercept) 4.467168 1.230107 3.632

Perspective2 -0.368816 0.212393 -1.736

Order2 0.021639 0.239935 0.090

ART 0.006492 0.034941 0.186

EQ 0.001132 0.010524 0.108

RH1 0.003150 0.181056 0.017

RH2 0.012498 0.163255 0.077

RH3 -0.002878 0.090363 -0.032

RH4 -0.008703 0.061073 -0.142

FantasyScaleIRI 0.027117 0.173580 0.156

Correlation of Fixed Effects:

(Intr) Prspc2 Order2 ART EQ RH1 RH2 RH3 RH4

Perspectiv2 -0.086

Order2 -0.304 0.000

ART -0.459 0.000 0.176

EQ -0.084 0.000 -0.311 -0.048

RH1 -0.533 0.000 0.168 0.152 -0.007

RH2 0.008 0.000 -0.003 0.256 -0.115 -0.688

RH3 -0.089 0.000 0.294 0.007 -0.164 0.100 -0.193

RH4 -0.074 0.000 -0.009 -0.226 0.218 0.126 -0.302 0.178

FntsySclIRI -0.718 0.000 0.254 0.264 -0.334 0.310 0.102 -0.047 -0.321

> PValues_Ranking<- Anova(modelFULLrank, type = 3, test = 'Chisq')

> PValues_Ranking

Analysis of Deviance Table (Type III Wald chisquare tests)

Response: Ranking

Chisq Df Pr(>Chisq)

(Intercept) 13.1880 1 0.0002817 ***

Perspective 3.0154 1 0.0824788 .

Order 0.0081 1 0.9281377

ART 0.0345 1 0.8525969

EQ 0.0116 1 0.9143052

RH1 0.0003 1 0.9861211

RH2 0.0059 1 0.9389773

RH3 0.0010 1 0.9745949

RH4 0.0203 1 0.8866858

FantasyScaleIRI 0.0244 1 0.8758600

---

Signif. codes: 0 ‘***’ 0.001 ‘**’ 0.01 ‘*’ 0.05 ‘.’ 0.1 ‘ ’ 1

> modelALLrank= lmer ( Ranking ~ Perspective + Order + EQ + ART + Rating + Immersion + PeaksD0.15 +(1|Subject)+(1|Story), data=ndata)

> summary(modelALLrank)

Linear mixed model fit by REML ['lmerMod']

Formula: Ranking ~ Perspective + Order + EQ + ART + Rating + Immersion + PeaksD0.15 + (1 | Subject) + (1 | Story)

Data: ndata

REML criterion at convergence: 1588.034

Random effects:

Groups Name Variance Std.Dev.

Subject (Intercept) 0.0000 0.0000

Story (Intercept) 0.2672 0.5169

Residual 3.4172 1.8486

Number of obs: 382, groups: Subject, 50; Story, 8

Fixed effects:

Estimate Std. Error t value

(Intercept) 1.361180 0.544627 2.499

Perspective2 -0.227567 0.192001 -1.185

Order2 -0.192514 0.194056 -0.992

EQ -0.017523 0.008724 -2.009

ART -0.057670 0.023816 -2.421

Rating 0.314692 0.075735 4.155

Immersion 0.592387 0.136202 4.349

PeaksD0.15 -0.008221 0.017780 -0.462

Correlation of Fixed Effects:

(Intr) Prspc2 Order2 EQ ART Rating Immrsn

Perspectiv2 -0.207

Order2 0.010 -0.005

EQ -0.470 -0.033 -0.131

ART -0.248 -0.013 0.004 0.218

Rating -0.102 -0.006 -0.034 0.039 -0.227

Immersion -0.324 0.080 -0.069 -0.211 0.024 -0.683

PeaksD0.15 -0.052 -0.117 -0.028 -0.114 -0.054 -0.032 -0.016

> PValues_Ranking<- Anova(modelALLrank, type = 3, test = 'Chisq')

> PValues_Ranking

Analysis of Deviance Table (Type III Wald chisquare tests)

Response: Ranking

Chisq Df Pr(>Chisq)

(Intercept) 6.2465 1 0.01244 *

Perspective 1.4048 1 0.23592

Order 0.9842 1 0.32117

EQ 4.0342 1 0.04459 *

ART 5.8635 1 0.01546 *

Rating 17.2655 1 3.250e-05 ***

Immersion 18.9167 1 1.366e-05 ***

PeaksD0.15 0.2138 1 0.64380

---

Signif. codes: 0 ‘***’ 0.001 ‘**’ 0.01 ‘*’ 0.05 ‘.’ 0.1 ‘ ’ 1

***EDA***

> modeld2slopes = lmer (PeaksD0.15 ~ Perspective + Order + (Perspective|Subject)+(Perspective|Story), data=ndata)

> summary(modeld2slopes)

Linear mixed model fit by REML ['lmerMod']

Formula: PeaksD0.15 ~ Perspective + Order + (Perspective | Subject) + (Perspective | Story)

Data: ndata

REML criterion at convergence: 2327.849

Random effects:

Groups Name Variance Std.Dev. Corr

Subject (Intercept) 8.8898 2.9816

Perspective 6.0045 2.4504 -0.45

Story (Intercept) 8.0914 2.8445

Perspective 0.3679 0.6065 1.00

Residual 13.9116 3.7298

Number of obs: 398, groups: Subject, 52; Story, 8

Fixed effects:

Estimate Std. Error t value

(Intercept) 5.6359 1.3306 4.235

Perspective 1.0392 0.5513 1.885

Order2 -1.4942 0.9606 -1.556

Correlation of Fixed Effects:

(Intr) Prspct

Perspective -0.085

Order2 -0.360 -0.001

> anova (modeld0, modeld1, modeld2, modeld3)

Data: ndata

Models:

modeld0: PeaksD0.15 ~ 1 + (1 | Subject) + (1 | Story)

modeld1: PeaksD0.15 ~ Order + (1 | Subject) + (1 | Story)

modeld2: PeaksD0.15 ~ Perspective + Order + (1 | Subject) + (1 | Story)

modeld3: PeaksD0.15 ~ Perspective + Order + Manipulated + (1 | Subject) +

modeld3: (1 | Story)

Df AIC BIC logLik deviance Chisq Chi Df Pr(>Chisq)

modeld0 4 2364.5 2380.5 -1178.3 2356.5

modeld1 5 2366.3 2386.2 -1178.2 2356.3 0.2017 1 0.653319

modeld2 6 2361.2 2385.1 -1174.6 2349.2 7.1338 1 0.007564 **

modeld3 7 2363.2 2391.1 -1174.6 2349.2 0.0159 1 0.899746

> anova (modeld2, modeld2slopes)

Data: ndata

Models:

modeld2: PeaksD0.15 ~ Perspective + Order + (1 | Subject) + (1 | Story)

modeld2slopes: PeaksD0.15 ~ Perspective + Order + (Perspective | Subject) +

modeld2slopes: (Perspective | Story)

Df AIC BIC logLik deviance Chisq Chi Df Pr(>Chisq)

modeld2 6 2361.2 2385.1 -1174.6 2349.2

modeld2slopes 10 2352.4 2392.3 -1166.2 2332.4 16.74 4 0.002171 **

> PValues_Peaks <- Anova(modeld2slopes, type = 3, test = 'Chisq')

> PValues_Peaks

Analysis of Deviance Table (Type III Wald chisquare tests)

Response: PeaksD0.15

Chisq Df Pr(>Chisq)

(Intercept) 22.8437 1 1.757e-06 ***

Perspective 3.5540 1 0.0594 .

Order 2.4197 1 0.1198

---

Signif. codes: 0 ‘***’ 0.001 ‘**’ 0.01 ‘*’ 0.05 ‘.’ 0.1 ‘ ’ 1

> modelEDA_FULL= lmer ( PeaksD0.15 ~ Perspective + Order + EQ + ART + (Perspective|Subject)+(Perspective|Story), data=ndata)

> summary(modelEDA_FULL)

Linear mixed model fit by REML ['lmerMod']

Formula: PeaksD0.15 ~ Perspective + Order + EQ + ART + (Perspective | Subject) + (Perspective | Story)

Data: ndata

REML criterion at convergence: 2285.873

Random effects:

Groups Name Variance Std.Dev. Corr

Subject (Intercept) 10.4514 3.2329

Perspective 6.2458 2.4992 -0.50

Story (Intercept) 7.3670 2.7142

Perspective 0.4284 0.6546 1.00

Residual 13.8305 3.7189

Number of obs: 390, groups: Subject, 51; Story, 8

Fixed effects:

Estimate Std. Error t value

(Intercept) 2.90262 2.37575 1.222

Perspective 1.06487 0.56587 1.882

Order2 -1.42920 0.99550 -1.436

EQ 0.04825 0.04364 1.106

ART 0.10130 0.11891 0.852

Correlation of Fixed Effects:

(Intr) Prspct Order2 EQ

Perspective -0.050

Order2 -0.065 0.001

EQ -0.764 -0.008 -0.187

ART -0.470 0.003 -0.030 0.193

> PValues_PeaksD0.15 <- Anova(modelEDA_FULL, type = 3, test = 'Chisq')

> PValues_PeaksD0.15

Analysis of Deviance Table (Type III Wald chisquare tests)

Response: PeaksD0.15

Chisq Df Pr(>Chisq)

(Intercept) 2.1062 1 0.14670

Perspective 3.8547 1 0.04961 *

Order 2.0099 1 0.15628

EQ 1.0402 1 0.30778

ART 0.5851 1 0.44432

Ranking 0.5534 1 0.45695

Immersion 0.0459 1 0.83042

Rating 0.0854 1 0.77006

---

Signif. codes: 0 ‘***’ 0.001 ‘**’ 0.01 ‘*’ 0.05 ‘.’ 0.1 ‘ ’ 1

> modelEDA_ALL= lmer ( PeaksD0.15 ~ Perspective + Order + EQ + ART + Ranking + Immersion + Rating +(Perspective|Subject)+(Perspective|Story), data=ndata)

> summary(modelEDA_ALL)

Linear mixed model fit by REML ['lmerMod']

Formula: PeaksD0.15 ~ Perspective + Order + EQ + ART + Ranking + Immersion + Rating + (Perspective | Subject) + (Perspective | Story)

Data: ndata

REML criterion at convergence: 2247.961

Random effects:

Groups Name Variance Std.Dev. Corr

Subject (Intercept) 10.5355 3.2458

Perspective 6.1536 2.4806 -0.49

Story (Intercept) 6.6120 2.5714

Perspective 0.6255 0.7909 1.00

Residual 14.1093 3.7562

Number of obs: 382, groups: Subject, 50; Story, 8

Fixed effects:

Estimate Std. Error t value

(Intercept) 2.53971 2.51683 1.009

Perspective 1.16859 0.59521 1.963

Order2 -1.44094 1.01631 -1.418

EQ 0.04557 0.04469 1.020

ART 0.09323 0.12188 0.765

Ranking 0.08512 0.11443 0.744

Immersion -0.08123 0.37930 -0.214

Rating 0.06145 0.21022 0.292

Correlation of Fixed Effects:

(Intr) Prspct Order2 EQ ART Rankng Immrsn

Perspective -0.053

Order2 -0.059 -0.002

EQ -0.689 -0.017 -0.161

ART -0.405 -0.003 -0.020 0.206

Ranking -0.015 0.041 0.042 0.040 0.049

Immersion -0.184 0.052 -0.059 -0.097 -0.007 -0.221

Rating -0.017 -0.022 0.003 -0.009 -0.122 -0.220 -0.648

> PValues_PeaksD0.15 <- Anova(modelEDA_ALL, type = 3, test = 'Chisq')

> PValues_PeaksD0.15

Analysis of Deviance Table (Type III Wald chisquare tests)

Response: PeaksD0.15

Chisq Df Pr(>Chisq)

(Intercept) 2.7002 1 0.10033

Perspective 3.5413 1 0.05986 .

Order 2.0610 1 0.15111

EQ 1.2225 1 0.26887

ART 0.7256 1 0.39431

---

Signif. codes: 0 ‘***’ 0.001 ‘**’ 0.01 ‘*’ 0.05 ‘.’ 0.1 ‘ ’ 1

##### Control model for duration

> model_duration = lmer (Duration ~ Perspective + Order + (Perspective|Subject)+(Perspective|Story), data=ndata)

> summary (model_duration)

Linear mixed model fit by REML ['lmerMod']

Formula: Duration ~ Perspective + Order + (Perspective | Subject) + (Perspective | Story)

Data: ndata

REML criterion at convergence: 9176.657

Random effects:

Groups Name Variance Std.Dev. Corr

Subject (Intercept) 830718788 28822

Perspective2 6240495 2498 1.00

Story (Intercept) 496009409 22271

Perspective2 7556634 2749 1.00

Residual 370156616 19239

Number of obs: 401, groups: Subject, 52; Story, 8

Fixed effects:

Estimate Std. Error t value

(Intercept) 35729 9875 3.618

Perspective2 1138 2191 0.519

Order2 3018 8428 0.358

Correlation of Fixed Effects:

(Intr) Prspc2

Perspectiv2 0.333

Order2 -0.426 -0.002

>

> PValues_model_duration <- Anova(model_duration, type = 3, test = 'Chisq')

> PValues_model_duration

Analysis of Deviance Table (Type III Wald chisquare tests)

Response: Duration

Chisq Df Pr(>Chisq)

(Intercept) 13.0903 1 0.0002968 ***

Perspective 0.2698 1 0.6034355

Order 0.1282 1 0.7203150

---

Signif. codes: 0 ‘***’ 0.001 ‘**’ 0.01 ‘*’ 0.05 ‘.’ 0.1 ‘ ’ 1
