# Supplementary material for: Taking Perspective: Personal Pronouns Affect Experiential Aspects of Literary Reading
Source: PLoS One. 2016 May 18;11(5):e0154732. doi: 10.1371/journal.pone.0154732 (PMC4883771; doi:10.1371/journal.pone.0154732)
Supplement: S1 Story — (DOCX) [file pone.0154732.s004.docx]

# S1 Story example A

# Officina Asmara

It is almost dark above the Ijsseldijk near Deventer. Heavy clouds press the light of the day from the world. 'I should have taken an umbrella with me after all', ***my/his*** father said. A jogger is catching up with ***us/them*** from behind on the bike path. ***I/He*** ***am/is*** always nervous when a jogger is running behind ***me/him***. It seems to ***me/him*** that they are plotting to steal something. In the distance along the dike are the silhouettes of cattle; Galloways imported from Scotland. Some of them calved in January already. (Once, on a winter's night, ***I/he*** was walking there with a loved one about the same dike, back then white with snow. ***We/They*** passed a herd of cattle who were breathing heavily. They lay close to each other, like dark hills. Streams of breath came out of their nostrils; a train was crossing the railroad bridge towards the west behind ***us/them***. ***We/they*** were deeply moved by the gentle beasts in the snow. The spring back then brought not only flowers and fresh grass, but also foot-and-mouth disease, so that the entire group had to be culled. Now there are new ones.)

***I/He offer/offers*** ***my/his*** father ***my/his*** cap to protect him against the first showers of rain brewing above ***us/them***, but he declines. ***We/they*** talk about news about the family of which he is the patriarch. 'Actually I think it’s a mess,' he says into the wind. 'Friends of mine have large family reunions several times a year, and more grandchildren than they can remember. And what do I have; two angry daughters, and a grandson who cannot listen. Not to mention you.' He hunches down in his collar. 'A serious relationship doesn't work for you, does it?'

***I/He*** remain***(s)*** silent. ***We/They*** turn back. As ***we/they*** arrive at ***my/his father's*** house he wants to show ***me/him*** something in the barn: an antique model of the Cutty Sark, the famous tea-clipper. The rigging is red, the wood is as decayed as a ship’s at the bottom of the sea. He says: 'Don't touch, I need to refurbish it first.'

Against the wall stands a table with a chair and piles of paper around it. ***I/He*** ***ask/asks*** what he is doing with all the piles of advertisements. 'An Eritrean asylum seeker does the sorting for his paper route here,' he says. Officina Asmara. When the Dutch team is playing, he always wears his orange cap'.

'So now you’re a criminal?'

Who gives a damn about the law?

Later that evening ***we/they*** sit at the kitchen table in the gray light. ***My/His*** father's face is full of shadows. ***I/He*** examine***(s)*** this dubious man, who refurbishes old ship models in his barn between piles of paper, and resolve***(s)*** to take a closer look as long as time still allows.
