# Supplementary material for: Taking Perspective: Personal Pronouns Affect Experiential Aspects of Literary Reading
Source: PLoS One. 2016 May 18;11(5):e0154732. doi: 10.1371/journal.pone.0154732 (PMC4883771; doi:10.1371/journal.pone.0154732)
Supplement: S2 Story — (DOCX) [file pone.0154732.s005.docx]

# S2 Story example B

# The Mexican dog

Mr. Kuisters from the fish shop, where ***I/he*** occasionally had to pick up sliced salmon for ***my/his*** mom on Friday afternoons after school, was a tall, bony man whose face consisted mainly of wrinkles. His head with its sheer ginger quiff was jutting out over his hunched shoulders. He cut the salmon with a thin knife that was worn in the middle. He took the slices between his thumb and index finger and carefully placed them side by side. His hands were purplish red and fish scales clung to them. He always asked ***me/him*** if **I/he** wanted to play with Tonia. ***I/He*** said ***I/he*** could not, because they were sitting at home waiting for the salmon.

But one day ***I/he*** could not escape. It was busy in the shop. As soon as Mr. Kuisters saw ***me/him*** come in he opened the sliding door between the shop and the apartment a bit and said that ***I/he*** should go inside because it would take quite a while. "Tonia is home." He pushed ***my/his*** shoulder and closed the door behind ***me/him***. The room was dark and small. Tonia sat at the table staring at her hands lying in front of her on the plush tablecloth; a pale child with light, watery eyes and hair like flax. She was in ***my/his*** class and because everyone thought she not only resembled a fish, but also reeked of fish, no one wanted to play with her.

"What are you doing?" she asked. She put her hands in her lap and looked at ***me/him*** suspiciously. Her mouth was half open; her white face gleamed like it was smeared with grease.

***"I have to wait here for your father."/He said that he has to wait for her father.***

The furniture in the room was placed so close together that you could hardly walk without touching something. ***I/He*** shoved the chair on which ***I/he*** leaned ***my/his*** forearms as far as possible under the table, but when ***I/he*** leaned back a bit ***I/he*** felt the key of the dresser in ***my/his*** back. The space was further cramped by a huge tasseled lamp that hung like a parasol over ***our/their*** heads. On the edge of the chimney stood a black metal pendulum clock with a little naked man on top of the pendulum. He held a kind of club in one hand and with the other he pointed down to the dial. The clock ticked loudly. And ***we/they*** didn't say a word to each other. About twenty minutes later her father came in.

"Well," he said. He locked the door with a hook and changed his white coat for a brown jacket that hung on a hanger in the closet. "Now we can go peacefully about our business." He pushed a couple of chairs aside, which was the only way to get to the small table in the corner of the room. On top of the table stood an apparatus with a front plate made of black ebonite. Coils emerged out of it and it had two buttons at the bottom with a white scale. Mr. Kuisters pressed down a lever on the side of the device, turned the knobs, put the coils in a particular position, and asked his daughter if there was a new fuse in the control box.

She nodded.

"Wonderful," he said, "then we can start." He looked at his watch. "It is just the right time. Come over here." He beckoned to ***me/him***, pulled a chair closer to the table and motioned to ***me/him*** that ***I/he*** should sit there. "Move your head slightly forward." He stood behind ***me/him*** and pushed gently against ***my/his*** crown, his hands went through ***my/his*** hair. A shiver crept down ***my/his*** spine up to ***my/his*** bottom. ***I/He*** smelled a sharp fishy smell that made ***me/him*** sick. The man put a double metal strap over ***my/his*** head and pressed two black discs with holes in them over ***my/his*** ears.

"Now listen," he cried, "here it comes." He leaned forward so that his big, saggy face hung in front of ***mine/him***. I/He saw the red veins in his watery eyes and how his pupils darted back and forth - he wanted to see what ***I/he*** was hearing. An immense noise filled ***my/his*** ears, cracking, wheezing, tearing screams, long whistles, all of which suddenly merged into a furious roar that echoed through ***my/his*** whole body. Mr. Kuisters laughed. Now, that is him," he shouted, "that's the Mexican dog."

***I/He*** tried to pull the discs from ***my/his*** ears, but he held them tight. He pressed his hand firmly on ***my/his*** head and turned one of the buttons.
"Here's HDO, the Hilversum Radio Broadcasting," someone shouted, and after a few unintelligible phrases some deafening music hit against ***my/his*** eardrums, as if ***my/his*** head was jammed in the horn of ***my/his*** father's gramophone.

After a while, Mr. Kuisters abruptly let go of the headphones. He didn't seem pleased with the outcome. Dazed, ***I/he*** remained seated in the chair.

"Move." Tonia pulled ***my/his*** arm. "It is my turn now."

With ringing ears and throbbing temples ***I/he*** got up and walked to the door. With some difficulty, ***I/he*** loosened the hook. The door rumbled back on its tracks. The fishy smell in the shop was stronger than ever. ***I/He*** only dared to breathe again once I was outside, and ***I/he*** was already half a block away before ***I/he*** noticed that ***I/he*** had forgotten the salmon.

One afternoon ***I/he*** came home from school, and while ***I/he*** hung ***my/his*** coat on the rack in the hallway, ***I/he*** heard someone talking very loudly. The sound came from the living room. There were no other voices talking. It remained a grim monologue. Presuming that there was a visitor with a very unfriendly manner of conversation, or one of ***my/his*** relatives revealing the truth about what they think of ***my/his*** family, I***/he*** gently opened the door and peeked inside.

Inside the room, ***my/his*** father and brother were standing on either side of the chimney. They had their heads slightly tilted and stared silently at the radio’s speakers.

"Who is shouting?" ***I/he*** asked.

"That's Hitler," ***my/his*** father said. He gave ***me/him*** a sign to be quiet.

***I/He*** remained there listening. It was only the first year that ***I/he*** had learned German at school, and ***I/he*** understood very little of it. ***I/He*** only understood the word "Juden", which the man uttered more and more often, in an increasingly contemptuous tone, as if he was kicking it. Even upstairs in ***my/his*** room ***I/he*** could hear his voice. His voice penetrated into every corner of the house. It even drowned out the sound of the rumbling sink faucet that ***I/he*** had opened to see whether ***I/he*** could still hear it against the running water.

***I/He*** arranged ***my/his*** books and notebooks, but before ***I/he*** started my homework, ***I/he*** climbed up the attic stairs. ***I/He*** closed the attic door behind ***me/him***. Without switching on the light ***I/he*** walked to the middle of the room and stood still. The sound of the voice was quieter here, but still very audible, and ***I/he*** went back to ***my/his*** room and started doing ***my/his*** homework. With ***my/his*** hands over ***my/his*** ears, ***I/he*** was trying to study ***my/his*** history lesson about the Holy Alliance. ***I/He*** felt the same sensation as years before at Mr. Kuisters’ when ***I/he*** had the hard discs of the headset over ***my/his*** ears and heard the sound of radio for the very first time. The Mexican dog. ***I/He*** pressed ***my/his*** hands firmly against **my/his** ears, as if ***I/he*** subconsciously felt what that voice would bring.
