# Supplementary material for: Taking Perspective: Personal Pronouns Affect Experiential Aspects of Literary Reading
Source: PLoS One. 2016 May 18;11(5):e0154732. doi: 10.1371/journal.pone.0154732 (PMC4883771; doi:10.1371/journal.pone.0154732)
Supplement: S3 Story — (DOCX) [file pone.0154732.s006.docx]

# S3 Story example C

# River

***Two people/We*** walk down the flood bank to the river***, a man and a woman/my wife and I***. ***They/We*** walk through the floodplains of the Lower Rhine, which drags itself reluctantly through its bed. The grass is flattened by the rain, tufts of sheep’s wool hang on the barbed wire.
After a while, ***they/we*** take a break. ***The man/I*** support(s) **my/his** hands on **my/his** knees and rest(s) in that bent position. Later ***they/we*** slowly proceed through the grass and the clay, which sticks on the soles of ***their/our*** shoe soles.
One of ***them/us*** is going to die soon. This is what the doctor told ***them/us***. ***They/We*** have been so aghast since that notice that ***they/we*** can only talk about trifles of a practical nature. Sometimes, ***he/I*** tries (try) something like: "We got pretty far, the two of us."
She would prefer it if ***he/I*** did not say such things, she thinks that it trivializes the seriousness of the matter. But she doesn't say it out loud. She nods and smiles.
Now that it has come to this, ***the man and the woman/my wife and I*** are suddenly separated from each other by different forms of loneliness; hers caused by the days she has left without **him/me**, ***his/mine*** caused by the days ***he/I*** will no longer have. In front of ***him/me*** waits an eternity that ***he/I*** will have to enter discarnate. ***He/I*** look(s) at the river, the clouds, ***his/my*** wife, and fear(s) the abyss in which philosophy and jazz no longer help. A literature quote crosses ***his/my*** mind; a line by Elias Canetti: "You're afraid of everything that does not come after death."
She thinks about the seven thousand hours in a day, the forty nine thousand days in a week, not to mention the light years of a month... She is so astonished by the time ***they/we*** spent together, just as if there would never be an end to that. So frivolous, so thoughtless. And in the back of her mind, she resents ***him/me***, and that ***he/I*** will leave her behind, alone. "The dying one takes the world along. Where to?" (Canetti, again).
In that way, ***the man and woman /my wife and I*** are walking to the river in different kinds of perpetuity.
