# Supplementary material for: Taking Perspective: Personal Pronouns Affect Experiential Aspects of Literary Reading
Source: PLoS One. 2016 May 18;11(5):e0154732. doi: 10.1371/journal.pone.0154732 (PMC4883771; doi:10.1371/journal.pone.0154732)
Supplement: S1 Story Summaries and Content Questions — (DOCX) [file pone.0154732.s007.docx]

# S1 Story summaries and content questions

### Officina Asmara

Synopsis: A grown man visits his father and is confronted with long-standing conflicts. He learns something unexpected about his father and realizes how little he actually knows about him.

*Content question excluded from threshold criteria.*What is the story about?

- *The relationship between parents and their grownup children*
- *Memories of a childhood*
- *Gaining peace of conscience*

### De tekening (The drawing)

A misunderstanding while ordering in a café causes the main character to remember an embarrassing episode from his childhood:
 As an 11 year old, he was invited to visit his best friend’s notorious uncle Wim. Uncle Wim was a diehard joker who had a reputation for his export quality cheese and his recreational swimming pool. In nervous anticipation of visiting the uncle, he prepared a present for him: a drawing of the swimming pool and the farm. When he handed over the present to uncle Wim, he was confronted with the fact that the swimming pool was in realty an old manure basin. The story ends in the café, where an unknown stranger comes to collect the main character and the two leave the scene.

What is uncle Wim best known for?

- Cow races
- Export quality cheese
- Freshly baked farmers bread
- Being highly talented at drawing

### Liberty Mountain

Synopsis: A married mother of 3 rants about skiing and tells funny anecdotes about her skiing experiences when she was younger. Unfortunately her husband and children are avid skiers and she is confronted with a skiing holiday every winter. While her children and husband are skiing, she visits a local replica of Lourdes Grotto and prays to the holy mother to let her learn skiing, too. On her way out of the monument she slips and sprains her ankle.

What did her parents always say about skiing?

- It is dangerous
- It is an appropriate exercise
- It is expensive, tiring and useless

### Dubbele tong (Double tong)

Kees and Simon live together and share everything until Kees is diagnosed with mouth cancer. Simon has to deal with the situation and especially being alone. At first he deviates from the daily routine and isolates himself. Then he starts to imitate Kees’ tongue- scraping habit and becomes obsessed with naming objects. After a visit to their local pub, he resolves to stop speaking at all. The story ends when he pierces his tongue with his chipped tooth.

*Content question excluded from threshold criteria.*
Which new habit does Simon get accustomed to when Kees is gone?

- *He starts drinking*
- *He becomes obsessed with words*
- *He befriends Willem*

### De vissers (The fisherman)

His cat is terminally ill, and he decides to relieve ‘Katman’ from his suffering. For the last day, he buys a fish and prepares it for the cat, but they run late for the vet, so the cat misses out on the fish. On the way to the vet, a series of remarkable incidents occur, and finally, he runs back home with the cat, where it finally gets its fish.

What did he buy at the market?

- Fresh vegetables to make soup
- A new travel basket for his cat
- Fish

### Rivier (River)

Synopsis: An elderly couple is on a walk across the Dutch dykes. During the story it is revealed that the man is terminally ill and some hints are given about how the couple deals with the situation from his perspective.

How does the couple deal with the situation?

- They talk a lot about it to help each other
- They try to do as many things together as possible
- Both are struggling on their own in silence.

### De Mexicaanse hond (The Mexican dog)

Synopsis: The story is situated in the 30s. Every Friday, the boy buys fish for his family in the local fish shop. The daughter of the owner is in his class. She does not have any friends and her father always encourages the boy to play with her. One day, he can’t come up with an excuse and ends up sitting with her for 20 minutes and not speaking a word. Her father then joins the party and lets the boy try his self-built radio with make-shift headphones. The boy, never having experienced the sound of radio before is upset and runs out of the house. Several years later his family owns a radio themselves and when the boy comes home from school he meets his family listening to one of the first broadcasts of Adolph Hitler. He experiences a similar sensation as back when he heard the sound of radio for the very first time.

Are the boy and the girl friends with each other?

- Yes, but secretly he has a crush on her
- No
- Yes, they have been friends for a long time.

### Broeder P. (Brother P.)

Brother P. has lived in an old Roman monastery for many years. He has a deep affection for butterflies and made a little flower garden to attract them. One day each year the brethren may take a day off service. This year he decided not to visit his family and instead to visit a zoo, famous for its butterfly garden. The day starts beautifully, but then a storm leaves him stranded on the roadside with a damaged motor, and he has to cancel his plans. The following winter he dies.

What happened to the car on his day off?

- A damaged motor
- Pushed off the road by the storm
- A traffic accident
